# Genome-wide association study reveals novel loci associated with body size and carcass yields in Shaoxing ducks

**DOI:** 10.1186/s12864-025-12411-1

**Published:** 2025-12-09

**Authors:** Wenwu Xu, Zhaobin Wang, Tao Zeng, Yong Tian, Tiantian Gu, Li Chen, Lizhi Lu

**Affiliations:** State Key Laboratory for Managing Biotic and Chemical Threats to the Quality and Safety of Agro-products, Key Laboratory of Livestock and Poultry Resources (Poultry) Evaluation and Utilization, Ministry of Agriculture and Rural Affairs, Institute of Animal Science & Veterinary, Zhejiang Academy of Agricultural Sciences, Hangzhou, 310021 PR China

**Keywords:** GWAS, Shaoxing duck, Body size, Carcass yield trait, Haplotype

## Abstract

Shaoxing ducks are a dual-purpose breed renowned for their delectable meat quality high egg-laying performance, and robust adaptability, leading to a growing market presence. Despite their economic significance, the genetic mechanisms influencing carcass growth in Shaoxing ducks remain largely unexplored. This study utilized genome-wide association studies (GWAS) and haplotype-sharing analysis to identify genomic regions and candidate genes associated with body size and carcass yield traits in this breed. Our investigation identified 50 genes associated with 18 body size and carcass yield traits. Notably, the *RSPO3* and *SPATA13* genes exhibited significant correlations with breast muscle weight (BMW) and leg muscle weight (LMW) traits. Additionally, *COL6A3* was significantly associated with eviscerated weight% (EWP) and half-eviscerated weight% (HEWP) traits. Genes such as *FGF9*, *LATS2*, and *PFKP*, known for their roles in muscle development and energy metabolism, may also influence LMW traits. Through linkage disequilibrium and haplotype-sharing analyses, we identified a haplotype on chromosome 3 significantly linked to LMW. These genes and the identified haplotype hold promise as candidate markers for molecular breeding programs aimed at improving carcass traits in Shaoxing ducks. Furthermore, we utilized a bootstrap test to validate the reliability of GWAS results derived from small experimental samples, enhancing the robustness of our findings. The methodology and code for this bootstrap test are accessible at https://github.com/xuwenwu248/bootstrap.sh/commit/07db59d129c24c384ba2c43f9bb3f8d62fce845b.

**Supplementary Information:**

The online version contains supplementary material available at 10.1186/s12864-025-12411-1.

## Introduction

Ducks have become an increasingly important source of meat globally, particularly in Asia, where they are consumed in various culinary forms [1–3]. This growing demand has spurred efforts to develop and breed a diverse range of duck varieties to meet shifting market preferences. The availability of diverse genetic resources is crucial for the successful development of improved duck breeds [4]. In-depth research into the genetic foundations of desirable traits within local duck populations provides a robust scientific basis for advancing genetic improvements in duck farming.

In 2005, Klein and colleagues conducted a pioneering GWAS that identified genetic variants near the Complement Factor H (*CFH*) gene locus associated with age-related macular degeneration. This seminal work underscored the efficacy of GWAS in elucidating genetic underpinnings of complex diseases [5]. Subsequently, high-throughput sequencing and GWAS methodologies have been increasingly employed in livestock research, including studies on ducks, to uncover candidate genes and mutations linked to traits such as body size and carcass yield [6, 7]. These approaches have led to the identification of significant quantitative trait loci (QTLs) and candidate genes across various local duck breeds, enhancing our understanding of the genetic architecture influencing these economically important traits [8].

Recent whole-genome analyses in Pekin ducks have identified 37 loci significantly associated with 17 carcass traits, implicating 37 candidate genes involved in various biological functions. Notably, a single nucleotide polymorphism (*SNP*) at position Chr1_140105435 (A > T) within the ATPase phospholipid transporting 11 A gene (*ATP11A*) has been linked to five weight-related traits. Additionally, genes such as *LOC101791418*, *TUBGCP3*, and *ATP11A* have been associated with key traits including 42-day body weight, eviscerated weight, half-eviscerated weight, and leg muscle weight% [9]. Another study identified *IGF2BP1* as a crucial gene influencing body size and feed efficiency [10]. Whole-genome resequencing of Jinding, Shanma, and Shaoxing duck breeds has highlighted growth-related genes such as *IGF1R* and skeletal development genes like *CDF5* [11]. Moreover, the *EIF2AK3* gene has been linked to body weight development across several local duck breeds [12]. Additionally, *CTDSPL* and *PKP1* have emerged as novel candidate genes for breast muscle thickness [13, 14]. Genotyping and genome-wide association studies have also identified two SNPs in the *AUTS2* gene that are related to leanness in Pekin ducks [15]. These findings provide valuable insights into the genetic mechanisms underlying growth and carcass traits in Pekin ducks, offering potential markers for selective breeding programs aimed at improving meat production and quality.

The Shaoxing duck is renowned for its early maturity, robust adaptability, well-developed chest and leg musculature, and flavorful meat, making it a prominent dual-purpose breed for both meat and egg production in China. Notably, its breeding population constitutes over 60% of the country’s total duck population. By leveraging its inherent advantages alongside the rapid growth and high egg production traits from other duck genetic resources, economically beneficial hybrid combinations can be achieved. In avian species, particularly chickens, *QTL* databases have documented over 1,500 *QTLs* associated with body weight traits. However, *QTLs* pertaining to specific body components, such as neck length, fossil bone length, and foot weight, remain scarce [16]. Moreover, research focusing on body size and carcass yield traits in ducks is limited, resulting in an unclear genetic foundation and a deficiency in molecular markers. This gap poses challenges for the application of hybrid breeding strategies. To address these limitations and support the advancement of duck breeding initiatives, it is imperative to conduct comprehensive studies that elucidate the genetic underpinnings of economically significant traits in ducks. Enhancing our understanding in this area will facilitate the development of precise molecular markers, thereby improving the efficiency and effectiveness of hybrid breeding programs.

## Materials and methods

### Ducks and phenotypes

This study involved 166 Shaoxing ducks sourced from Hubei Shendan Co., Ltd. All specimens originated from a uniform purebred cohort of the same generation and were individually housed in three-tiered cages. The facility was equipped with wet curtain cooling systems, nipple drinkers, and automated feeders to ensure consistent daily nourishment. At 66 weeks of age, the ducks were humanely slaughtered following standard commercial protocols. Subsequently, eighteen morphometric and carcass yield parameters were assessed in accordance with the poultry performance terminology and measurement statistics outlined in the Ministry of Agriculture of China’s NY/T 823–2020 standard. These traits included body diagonal length (BDL), fossil bone length (FBL), breast depth (BD), breast width (BW), Shank Length (SL), Shank circumference (SC), Pelvis width (PW), Slaughter Weight (SW), Dressed Weight (DW), Dressed percentage (DP), Eviscerated Weight (EW), Eviscerated weight% (EWP), Half-eviscerated weight (HEW), Half-eviscerated weight% (HEWP). Breast muscle weight (BMW), Breast muscle weight% (BMWP), Leg muscle weight (LMW), Leg muscle weight% (LMWP) [17]. The following traits were measured using a caliper and weighing scale and calculated as follows:

- BDL: Distance measurement from the shoulder joint to the ischial tuberosity on the body surface (cm).
- FBL: The distance between the anterior and the posterior border of the breast-bone crest (cm).
- BD: The distance from the first thoracic vertebra to the anterior edge of the fossil bone (cm).
- BW: The distance vertically between the backbone and the beginning of the breast-bone crest (cm).
- SL: Measured with calipers as the straight-line distance from the upper joint of the tibia to the midpoint between the third and fourth toes (cm).
- SC: Circumference of the middle part of the shank (cm).
- PW: The distance between the two hip bone nodes (cm).
- SW: The weight of the duck after 6 h of fasting before slaughter (kg).
- DW: Weight after bloodletting and removal of feathers, foot cuticles, toes, and clam shells (kg).
- DP: DW/SW × 100%.
- EW: Half-eviscerated weight minus heart, liver, proventriculus, gizzard, and abdominal fat (kg).
- EWP: EW/SW × 100%.
- HEW: Weight of carcass after removal of trachea, esophagus, crop, intestines, spleen, pancreas, gallbladder, reproductive organs, stomach contents, and keratinocytes (kg).
- HEWP: HEW/SW × 100%.
- BMW: Incise the skin along the sternum ridge and dissect it towards the dorsal side. Use a scalpel to sever the muscles and tendons attached to the lateral surfaces of the sternum ridge and scapula. Then, carefully separate the entire skinless pectoral muscle and weigh it (g).
- BMWP: BMW/EW×100%.
- LMW: The weight of the total leg muscle after removal of the bones, skin, and subcutaneous fat (g).
- LMWP: LMW/EW×100%.

The normality of the evaluated traits was assessed using the Shapiro-Wilk test, a standard method for determining the normality of data distributions. For traits exhibiting significant deviations from normality, rank transformation was applied to normalize them for the mixed linear model [9, 18]. The degrees of freedom for the data analysis in this study were 160, and the corresponding critical r values are ± 0.154 and ± 0.200 at the 0.05 and 0.01 significance levels, respectively. Therefore, this study used these thresholds to determine significance when comparing traits. All experimental ducks were sampled and measured for relevant phenotypes after being euthanized by carotid artery bleeding. All procedures involving the care and use of ducks adhered strictly to ethical guidelines and were approved by the Institutional Animal Care and Use Committee (IACUC) of the Zhejiang Academy of Agricultural Sciences (Approval No. 2021 ZAASLA15).

### Genome sequencing

Genomic DNA was isolated from blood samples employing a modified cetyltrimethylammonium bromide (CTAB) protocol. The integrity and concentration of the extracted DNA were evaluated using agarose gel electrophoresis. For each qualified DNA sample, paired-end libraries were constructed following established protocols. This process included the fragmentation of genomic DNA to achieve an average insert size of approximately 500 base pairs (bp). The fragmented DNA underwent end repair to generate blunt ends, addition of a single adenine (A) nucleotide to the 3’ ends (A-tailing), and ligation of paired-end adaptors. These adaptors are essential for hybridization to the flow cell during sequencing. Subsequently, polymerase chain reaction (PCR) amplification was performed to selectively enrich DNA fragments that had adaptor molecules on both ends, ensuring the amplification of the desired fragments for sequencing. The prepared libraries were subjected to 150-bp paired-end sequencing on an Illumina HiSeq platform, adhering to the manufacturer’s guidelines. Paired-end sequencing involves reading each DNA fragment from both ends, which enhances the accuracy of read alignment and facilitates the detection of genomic rearrangements, insertions, deletions, and novel transcripts. The 150-bp read length provides a balance between data quality and the ability to map reads to the reference genome effectively. An average sequencing depth of 10× was achieved across all experimental samples. This depth ensures reliable variant detection and genotyping, meeting the requirements for comprehensive population genetic analyses [19].

### Variant discovery and genotyping

Raw sequencing reads were aligned to the CAU duck 1.0 reference genome using Burrows-Wheeler Aligner (BWA) [20]. Approximately 96.4% of the reads were successfully aligned, achieving an average sequencing depth of ×10 (range: ×8 to ×18) per sample. Detailed mapping information for the resequenced samples is available in Additional file 1: Table S1, Table S2, and Table S3. Duplicate paired-end reads mapping to the same reference position were identified and removed using Picard’s MarkDuplicates tool to minimize potential biases in variant detection. Subsequently, raw variants were called at individual bases using GATK’s HaplotypeCaller (version 4.0) [21], generating 166 individual ‘*.gvcf’ files. These files underwent joint variant calling with GATK’s GenotypeGVCFs tool. Variant quality control was performed using GATK’s VariantFiltration tool, applying the following filter expression for single nucleotide polymorphisms (SNPs): QD < 2.0 || FS > 60.0 || MQ < 40.0. SNPs were excluded based on the following criteria: (1) minor allele frequency > 0.05; (2) maximum missing rate < 0.1; and (3) only two alleles. The remaining SNPs were annotated using SnpEff, utilizing the gene annotations from the reference genome [22].

### Single-Trait GWAS analysis

The single-marker association between the genetic variants and the phenotype was assessed using GEMMA (v.0.94) based on a univariate linear mixed model (refer to Eq. 1), as outlined in the following equation [23]:

1 $$\begin{aligned}\mathrm{y} = &\mathrm{x} \beta\: + \: u + \: \epsilon; \:\mathrm{u}\:\sim \: {MVN}_{n}\left(0,\lambda\:{\tau\:}^{-1}\mathrm{K}\right),\\&\:\epsilon\:\sim\:{MVN}_{n}\left(0,{\tau\:}^{-1}{\mathrm{I}}_{n}\right)\end{aligned}$$

The experiments exclusively utilized female ducks, sourced from consistent breeding farms to minimize environmental variability. Ducks were grouped based on age to control for developmental differences. All subjects underwent slaughtering in synchronized batches to ensure uniformity. Consequently, gender and batch were not incorporated as fixed effects in the GEMMA model. Phenotypic data, represented by the vector y, encompassed the observed traits, including a column of ones to account for the intercept in statistical analyses. Genotypic data, denoted as the vector x, consisted of the genotypes at each locus under investigation; *β* is the effect of *SNPs*; u is a vector of random effects following the multivariate normal distribution $\:{MVN}_{n}\left(0, \lambda\:{\tau\:}^{-1}\mathrm{K}\right)$, in which λ the ratio of the additive genetic variance to the variance of residual variance, $\:{{\uptau\:}}^{-1}$ is the variance of the residual errors, and K is a kinship matrix estimated from whole-genome sequence variants; $\epsilon$ is a vector of errors following the multivariate normal distribution (see Eq. 1) and $\:{\mathrm{I}}_{n}$ is an identity matrix. To reduce the false positive rate, conservative Bonferroni correction method was used to obtain the thresholds for genome-wide significance and chromosome-wide significance, which are *P* = 0.05/N and *P* = 1/N respectively, where N is the number of effective SNP loci [24]. The validity of genome-wide association studies is affected by population stratification, which is one of a variety of influences [25]. Population stratification can substantially affect the accuracy of genome-wide association studies (GWAS) by introducing confounding factors that may lead to spurious associations. To address this issue, we utilized PLINK to assess the population structure within our study cohorts [26]. Additionally, we employed R software (v3.6.1) to generate quantile-quantile (Q-Q) plots, a standard method for detecting population stratification in GWAS analyses [25]. We conducted a Principal Component Analysis (PCA) to assess the genetic similarity among the experimental ducks using Plink software (version 1.9). The statistical power of the GWAS was calculated using GCTA software, incorporating the following parameters: sample size, heritability, Type 1 error rate for power estimation, and the variance in genetic relationships derived from single nucleotide polymorphisms (*SNPs*) ($\:{\uplambda\:}{{\uptau\:}}^{-1}$).

### Calculation of phenotypic variation explained by QTL

This study employed PLINK to filter loci within QTL regions by linkage disequilibrium (LD). Specifically, for SNPs showing pairwise LD values greater than 0.4, only one representative SNP was retained, whereas all SNPs with LD ≤ 0.4 were kept. The proportion of phenotypic variance explained (PVE) by significant SNPs was then estimated using the formula:

$$\:PVE=\frac{{v}_{\mathrm{residualReduced}}-{v}_\mathrm{residualFull}}{{v}_{\mathrm{residualReduced}}}$$

Where VresidualFull and VresidualReduced represent denote the residual variances from GWAS models fitted without and with the associated SNP loci, respectively.

### Post GWA analysis

In this study, we identified key *QTLs* through linkage disequilibrium (LD) analysis. We examined associations between significant *SNPs* and neighboring *SNPs*, adopting an LD decay threshold where r² decreases to approximately 0.4 across the poultry genome. This threshold selection aligns with established practices in poultry genetics research to ensure robust marker-trait associations. Candidate genes within these significant *QTL* regions were subsequently identified to perform GO based on biological process analysis in DAVID (available at http://david.abcc.ncifcrf.gov/home.jsp).

We identified the lead variant as the marker exhibiting the smallest *P*-value within each *QTL*. To assess LD between the lead variant and other variants, we employed PLINK (version 1.9) using the --r^2^ command, which calculates the squared correlation coefficient (r²) between pairs of variants [25]. Subsequently, we utilized fastPHASE with default parameters to construct haplotypes within the defined confidence intervals. Each haplotype was thoroughly examined to identify shared susceptibility haplotypes [27].

### Functional enrichment (GO and KEGG)

Candidate genes prioritized from the GWAS for body size and carcass yield were subjected to analysis.

### Bootstrap test

In our study, we utilized the bootstrap resampling technique to evaluate the robustness of our GWAS findings. Bootstrapping involves generating multiple resampled datasets from the original sample by sampling with replacement, allowing for the estimation of the sampling distribution of a statistic. This method facilitates the calculation of standard errors, confidence intervals, and hypothesis testing. Specifically, we created 1,000 bootstrap samples, each with the same number of subjects as the original sample (166), are generated via re-sampling with replacement. Then we conducted GWAS analysis on the newly composed 166 individuals to determine if the vulnerable regions identified in our study still exhibited strong signals, and finally counted how many times significant signals were identified in the 1000 GWAS experiments.

## Results

### Phenotype and genetic parameter statistics

**Fig. 1:**
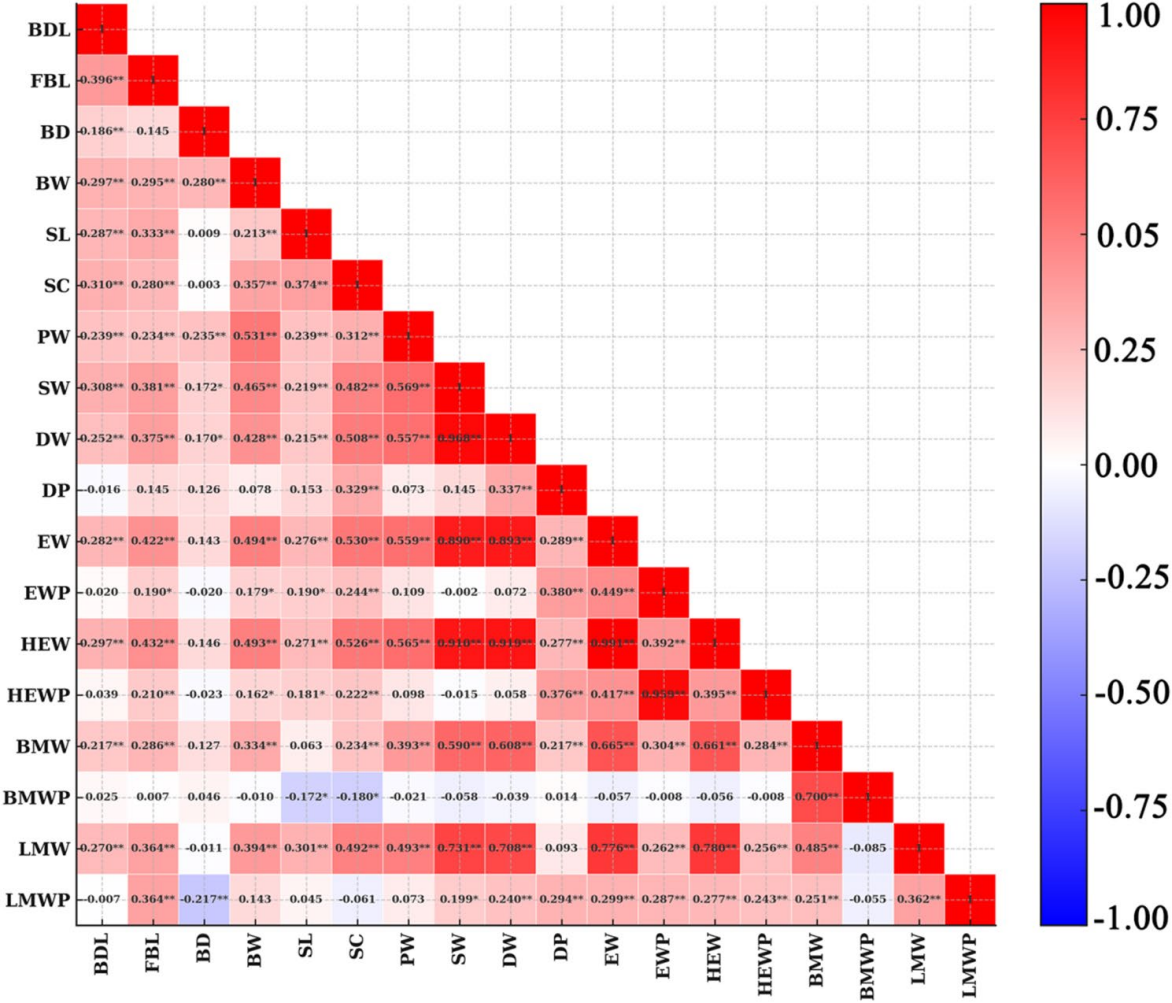
The phenotypic correlations between eighteen traits. The colors (numbers) represent the pairwise correlation coefficients of the serum biochemical indicators. Red indicates a positive correlation, and Blue indicates a negative correlation. ** and * indicate extremely significant and significant, respectively

Figure S1 in Additional File 1 demonstrates that the eighteen phenotypes conform to a normal distribution. Table 1 presents the descriptive statistics for these traits. Phenotypic correlations among the traits are displayed in Table 2; Fig. 1, with SNP-based heritability estimates shown on the diagonal in Table 2. The heritability of FBL (0.33), BD (0.21), and HEWP (0.21) was moderate, while BDL (0.16), SL (0.17), SC (0.17), EWP (0.13), HEW (0.11), BMW (0.15), BMWP (0.19), DP (0.12) and LMW (0.10) were traits with low heritability. In contrast, the heritability of BW, SW, DW, PW, LMWP, and EW was less than 0.1. Correlation analysis of phenotypes showed that EW, SW, DW, and HEW were all significantly and positively correlated with each other, with correlation coefficients greater than 0.890. In addition, there was also an extremely significant positive correlation between HEWP and EWP (*r* = 0.959**), suggesting that indirect selection can be applied when breeding for these traits. The correlations between many traits were very low; for example, the correlations of BDL with DP, HEWP, and BMWP were below 0.050. Our study also found that the degrees of negative correlation between traits were generally low. LMWP was significantly negatively correlated with SW, DW, DP, EW, EWP, HEW, HEWP, and BMW, but the correlation coefficients were all less than 0.30. These findings are consistent with the results obtained by Dend et al. in Pekin ducks [13]. The specific correlations among traits are shown in Table 2; Fig. 1.

**Table 1:** Descriptive statistics of phenotypic traits

Traits	*N*	Min	Max	Mean	SD
BDL (cm)	166	18.70	23.60	20.77	0.94
FBL (cm)	166	9.40	12.10	10.75	0.54
BD (cm)	166	5.60	7.20	6.36	0.30
BW (cm)	166	6.50	8.20	7.40	0.31
SL (cm)	166	4.60	6.40	5.70	0.26
SC (cm)	166	3.00	4.30	3.39	0.17
PW (cm)	166	5.50	7.10	6.30	0.31
SW (kg)	166	1.10	2.30	1.57	0.19
DW (kg)	166	0.96	2.02	1.39	0.18
DP (%)	166	0.82	0.97	0.88	0.02
EW (kg)	166	0.69	1.50	0.95	0.13
EWP(%)	166	0.49	0.72	0.60	0.04
HEW(kg)	166	0.77	1.63	1.05	0.14
HEWP(%)	166	0.56	0.79	0.67	0.04
BMW(g)	166	49.98	180.70	111.93	21.09
BMWP(%)	166	0.07	0.17	0.12	0.02
LMW(g)	166	67.74	138.40	94.88	13.35
LMWP(%)	166	0.08	0.12	0.10	0.01

*BDL* body diagonal length, *FBL* fossil bone length, *BD* breast depth, *BW* breast width, *SL* shank length, *SC* shank circumference, *PW* pelvis width, *SW* slaughter weight, *DW* dressed weight, *DP* dressed percentage, *EW* eviscerated weight, *EWP* eviscerated weight%, *HEW* half-eviscerated weight, *HEWP* half-eviscerated weight%, *BMW* breast muscle weight, *BMWP* breast muscle weight%, *LMW* leg muscle weight, *LMWP* leg muscle weight%. N, number of samples; Min, minimum phenotypic value; Max, maximum phenotypic value; SD, standard deviation

**Table 2:** *SNP*-based heritability and phenotypic correlations among 18 traits

Traits	BDL	FBL	BD	BW	SL	SC	PW	SW	DW	DP	EW	EWP	HEW	HEWP	BMW	BMWP	LMW	LMWP
**BDL**	0.16																	
**FBL**	0.396^**^	0.33																
**BD**	0.186^*^	0.145	0.21															
**BW**	0.297^**^	0.295^**^	0.280^**^	0.09														
**SL**	0.287^**^	0.333^**^	0.009	0.213^**^	0.17													
**SC**	0.310^**^	0.280^**^	0.003	0.357^**^	0.374^**^	0.17												
**PW**	0.239^**^	0.234^**^	0.235^**^	0.531^**^	0.239^**^	0.312^**^	0.06											
**SW**	0.308^**^	0.381^**^	0.172^*^	0.465^**^	0.219^**^	0.482^**^	0.569^**^	0.09										
**DW**	0.252^**^	0.375^**^	0.170^*^	0.428^**^	0.215^**^	0.508^**^	0.557^**^	0.968^**^	0.08									
**DP**	-0.016	0.145	0.126	0.078	0.153^*^	0.329^**^	0.073	0.145	0.337^**^	0.12								
**EW**	0.282^**^	0.422^**^	0.143	0.494^**^	0.276^**^	0.530^**^	0.559^**^	0.890^**^	0.893^**^	0.289^**^	0.09							
**EWP**	0.020	0.190^*^	-0.020	0.179^*^	0.190^*^	0.244^**^	0.109	-0.002	0.072	0.380^**^	0.449^**^	0.13						
**HEW**	0.297^**^	0.432^**^	0.146	0.493^**^	0.271^**^	0.526^**^	0.565^**^	0.910^**^	0.919^**^	0.277^**^	0.991^**^	0.392^**^	0.11					
**HEWP**	0.039	0.210^**^	-0.023	0.162^*^	0.181^*^	0.222^**^	0.098	-0.015	0.058	0.376^**^	0.417^**^	0.959^**^	0.395^**^	0.21				
**BMW**	0.217^**^	0.286^**^	0.127	0.334^**^	0.063	0.234^**^	0.393^**^	0.590^**^	0.608^**^	0.217^**^	0.665^**^	0.304^**^	0.661^**^	0.284^**^	0.15			
**BMWP**	0.025	0.007	0.046	-0.010	-0.172^*^	-0.180^*^	-0.021	-0.058	-0.039	0.014	-0.057	-0.008	-0.056	-0.008	0.700^**^	0.19		
**LMW**	0.270^**^	0.364^**^	-0.011	0.394^**^	0.301^**^	0.492^**^	0.493^**^	0.731^**^	0.708^**^	0.093	0.776^**^	0.262^**^	0.780^**^	0.256^**^	0.485^**^	-0.085	0.11	
**LMWP**	-0.007	-0.065	-0.217^**^	-0.143	0.045	-0.061	-0.073	-0.199^*^	-0.240^**^	-0.294^**^	-0.299^**^	-0.287^**^	-0.277^**^	-0.243^**^	-0.251^*^	-0.055	0.362^**^	0.02

Values on the diagonal are SNP-based heritability estimates; values below the diagonal are phenotypic correlation coefficients. ** and * indicate extremely significant and significant, respectively

### Genome-wide association study

**Fig. 2:**
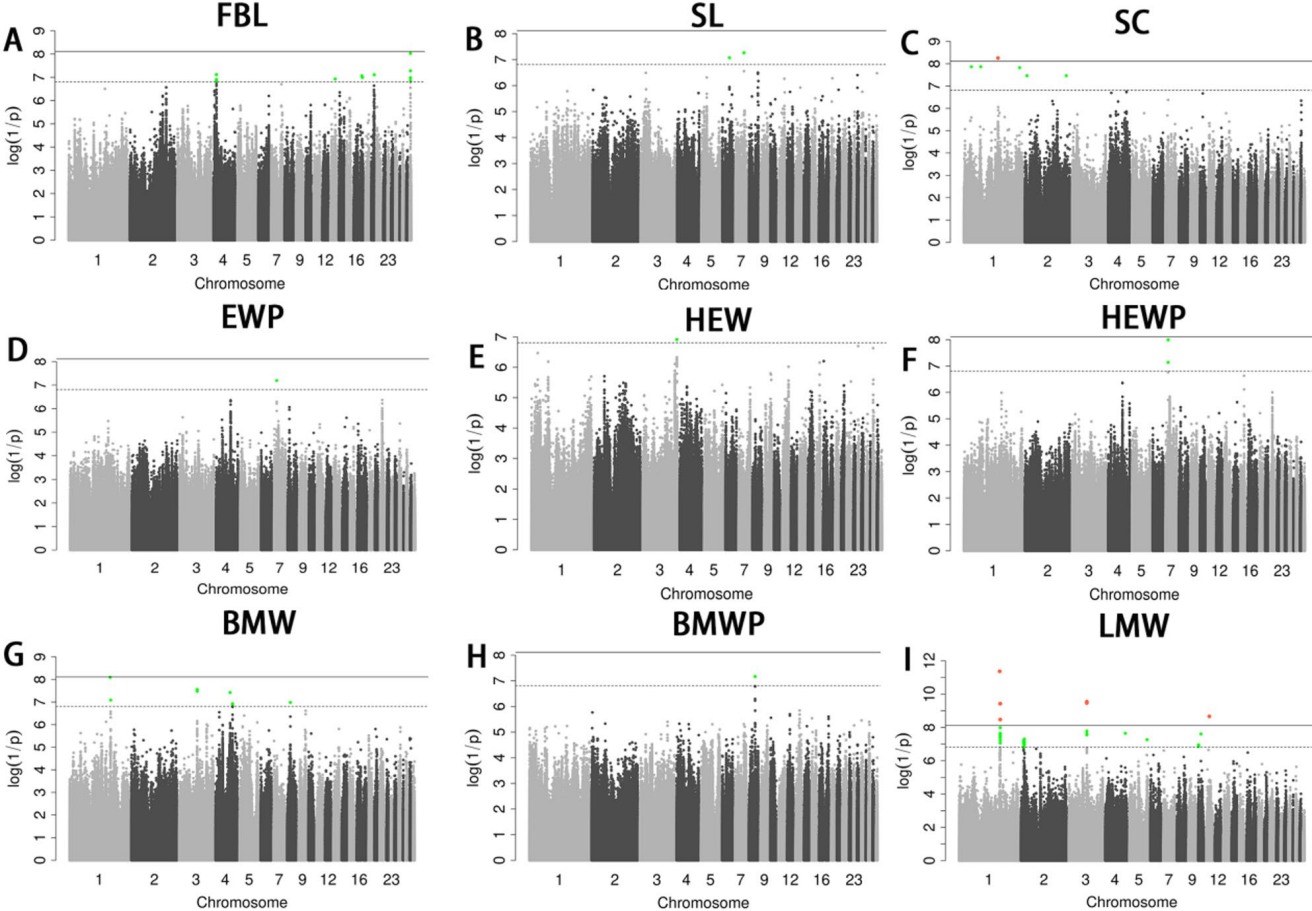
Manhattan plots derived from GWASs for FBL (**A**), SL (**B**), SC (**C**), EWP (**D**), HEW (**E**), HEWP (**F**), BMW (**G**), BMWP (**H**) and LMW (**I**). The y-axis and x-axis in this diagram, respectively, stand for the genomic coordinates split by chromosomes and the negative log10 P value of SNPs. Each dot on the diagram corresponds to an SNP found in the dataset. Black solid lines indicated the 5 percent genome-wide Bonferroni-corrected threshold, while tomato puree patches showed SNPs that exceed the chromosome-wide significance requirement

**Fig. 3:**
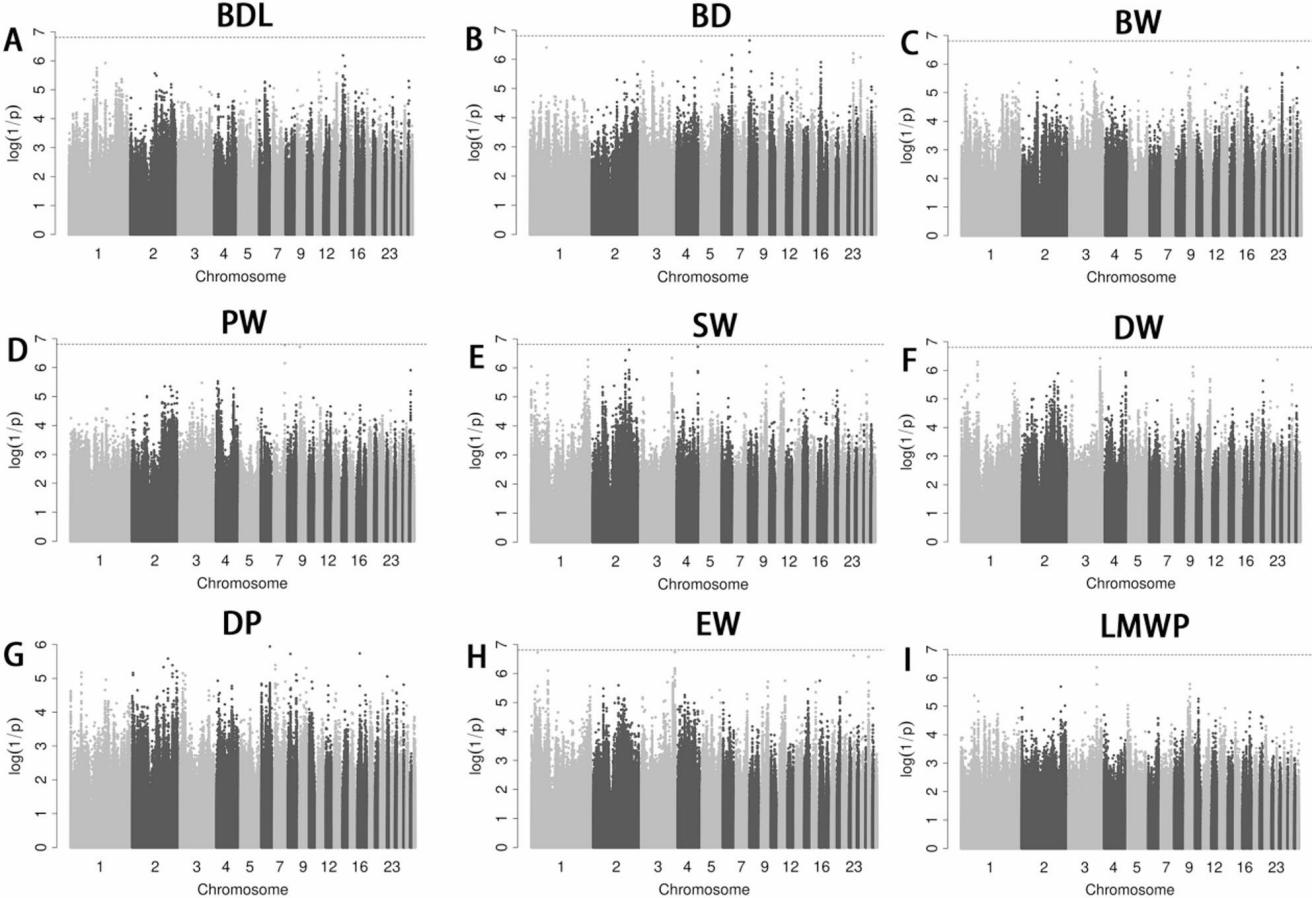
Manhattan plots showing associations of all SNPs with BDL (**A**), BD (**B**), BW (**C**), PW (**D**), SW (**E**), DW (**F**), DP (**G**), EW (**H**) and LMWP (**I**). In Manhattan plots, SNPs are plotted on the x-axis according to their position on each chromosome, against association with these traits on the y-axis (shown as − log10P-value). Black dashed line indicates chromosome-wide significance association (*P* = 1.48E − 07)

After quality control, a total of 6,746,746 *SNPs* and 166 individuals were retained. GWAS for all eighteen phenotypes were performed using a univariate linear model with genome-wide and chromosome-wide thresholds of 7.41E-09 and 1.48E-07, respectively. No loci exceeded the chromosome-wide threshold for BDL, BD, BW, PW, SW, DW, DP, EW and LMWP (Fig. 3A-I). For FBL, we detected signals on chromosomes 4, 13, 18, 20, and 29; the lead SNP on chromosome 29 (29_2451533, *P*-value = 9.37E-09) approached the genome-wide level, and an additional QTL was observed on chromosome 4 (~ 10.06–10.11 Mb), with the most significantly associated *SNP* being 4_10113655 (*P*-value = 7.64E-08) (Fig. 2A; Table 3). Two SNPs exceeded the chromosome-wide significance level for the trait SL on chromosome 6 and 7 (Fig. 2B; Table 3). On chromosome 7, (~ 9.09 Mb), two adjacent SNPs were associated with HEWP and the region also harbored the top signal for EWP, whereas HEW showed one locus on chromosome 3. BMW presented 11 SNPs across chromosomes 1, 3, 4 and 8, including a major region on chr1 (~ 137.43–139.27 Mb) and a QTL on chr3 (~ 61.13–61.17 Mb). LMW displayed the strongest evidence with 8 genome-wide and 38 chromosome-wide significant SNPs, highlighting the same chromosome 1 region (~ 137.43–139.29 Mb) and a chromosome 3 locus (~ 61.13–61.23 Mb). In total, 53 candidate genes were prioritized within ± 0.5 Mb of significant SNPs (Tables 3 and 4).

**Table 3:** Significant *SNPs* associated with FBL, SL, HEWP, EWP, HEW, BMWP and BMW

Trait	Chr	Position	PVE	P_wald	Candidate genes
FBL	4	10,113,655	1.64%	7.64E-08	*CISD2* *UBE2D3*
	4	10,063,953		1.24E-07	
	4	10,113,379		1.45E-07	
	13	16,111,870	1.79%	1.17E-07	*SUCLG2*
	18	6,204,837	1.60%	8.64E-08	*EGFL7*
	18	8,525,466		1.01E-07	
	20	10,290,550	1.71%	7.75E-08	*VEZF1*,* CUEDC1*
	29	2,451,533	1.81%	9.37E-09	*DPP9* *TNFAIP8L1*
	29	2,442,567		5.26E-08	
	29	2,454,510		1.04E-07	
	29	2,440,813		1.39E-07	
SL	6	23,789,860	3.21%	8.46E-08	*PCGF6*
	7	29,103,961	2.36%	5.46E-08	*SMAFCAL1*,* MARCHF4*
HEWP	7	9,092,892	3.19%	1.01E-08	*COL6A3*
	7	9,093,132		7.25E-08	*CPPS8*
EWP	7	9,092,892	1.59%	6.39E-08	*COL6A3*
HEW	3	114,147,240	1.58%	1.20E-07	*XKR6*
BMWP	8	21,296,962	1.68%	6.81E-08	*ST3GAL3*,* KDM4A*
BMW	1	137,437,762	2.55%	8.01E-09	*GJA3*
	1	139,276,950		8.16E-08	*SPATA13*
	3	61,179,349	4.77%	2.77E-08	*RSPO3*
	3	61,134,622		3.32E-08	
	4	49,527,971	1.36%	3.78E-08	*PRDM5*
	4	57,865,980		1.18E-07	*GAB1*
	4	57,802,028		1.41E-07	*SMARCA5*
	8	11,109,180	1.53%	1.04E-07	*TOR1AIP2*

*Chr* chromosome number, Position: base positions on the chromosome, *P_wald* P-value from the wald test, *PVE* Phenotypic variance explained by significant QTL

**Table 4:** Significant SNPs associated with LMW and SC

Trait	Chr	Position	PVE	P_wald	Candidate genes
LMW	1	137,437,762	1.36%	4.34E-12	*CRYL1* *IFT88* *IL17D* *XPO4* *EEF1AKMT1* *LATS2* *MRPL57* *MICU2* *FGF9* *SGCG* *SACS* *TNFRSF19* *MIPEP* *SPATA13*
	1	139,294,407		3.77E-10	
	1	139,296,791		3.77E-10	
	1	139,276,950		3.80E-10	
	1	139,249,357		3.39E-09	
	1	139,267,854		1.03E-08	
	1	139,268,302		1.05E-08	
	1	139,279,244		2.25E-08	
	1	139,255,969		2.70E-08	
	1	139,256,948		2.70E-08	
	1	139,276,997		2.87E-08	
	1	139,293,680		3.31E-08	
	1	139,250,184		3.67E-08	
	1	139,297,030		4.23E-08	
	1	139,234,431		6.00E-08	
	1	139,238,602		8.76E-08	
	2	12,054,090	6.12%	7.64E-08	*PFKP* *PITRM1*
	2	12,056,322		5.03E-08	
	2	12,060,151		1.04E-07	
	2	9,441,955	3.45%	6.73E-08	
	2	9,442,602		1.40E-07	
	2	9,442,825		7.75E-08	
	2	9,443,165		1.40E-07	
	2	9,526,711		5.96E-08	
	2	9,526,934		9.50E-08	
	2	9,530,548		7.80E-08	
	3	61,179,349	3.40%	2.88E-10	*RSPO3* *RNF146* *ECHDC1*
	3	61,134,622		3.42E-10	
	3	61,229,580		1.67E-08	
	3	61,180,035		2.39E-08	
	3	61,179,992		2.84E-08	
	3	61,180,026		2.84E-08	
	4	68,383,137	3.55%	2.27E-08	*GALNT7*,* HMGB2*
	5	59,938,431	1.20%	5.48E-08	*OTX2*
	10	9,676,251	1.48%	2.47E-08	*ZMYM3*,* GJB1* *NONO* *TBX22*,* CHMP1B*
	10	1,732,490	5.30%	1.12E-07	
	10	1,146,302	0.10%	1.40E-07	
	11	10,314,100	2.60%	2.19E-09	*AP3B2*
SC	1	116,995,667	1.70%	5.57E-09	*NUP37*
	1	25,375,335	1.70%	1.37E-08	
	1	57,751,928	1.78%	1.37E-08	
	1	191,506,544	1.63%	1.50E-08	
	2	142,581,128	1.10%	3.43E-08	/
	2	7,262,667	1.10%	3.43E-08	

*Chr* chromosome number; position: base positions on the chromosome, *P_wald* P-value from the wald test, *PVE* Phenotypic variance explained by significant QTL

### Assessment of population stratification

To assess potential population stratification, we examined QQ plots from eighteen genome-wide analyses of body size and carcass yield traits (Additional file 1: Figure S2). The genomic inflation factor (λ) ranged from 0.94 to 1.03. A PCA of all the Ducks has revealed that there is no distinct population stratification among the population (Additional file 1: Figure S3).

### Post GWA analysis

**Fig. 4:**
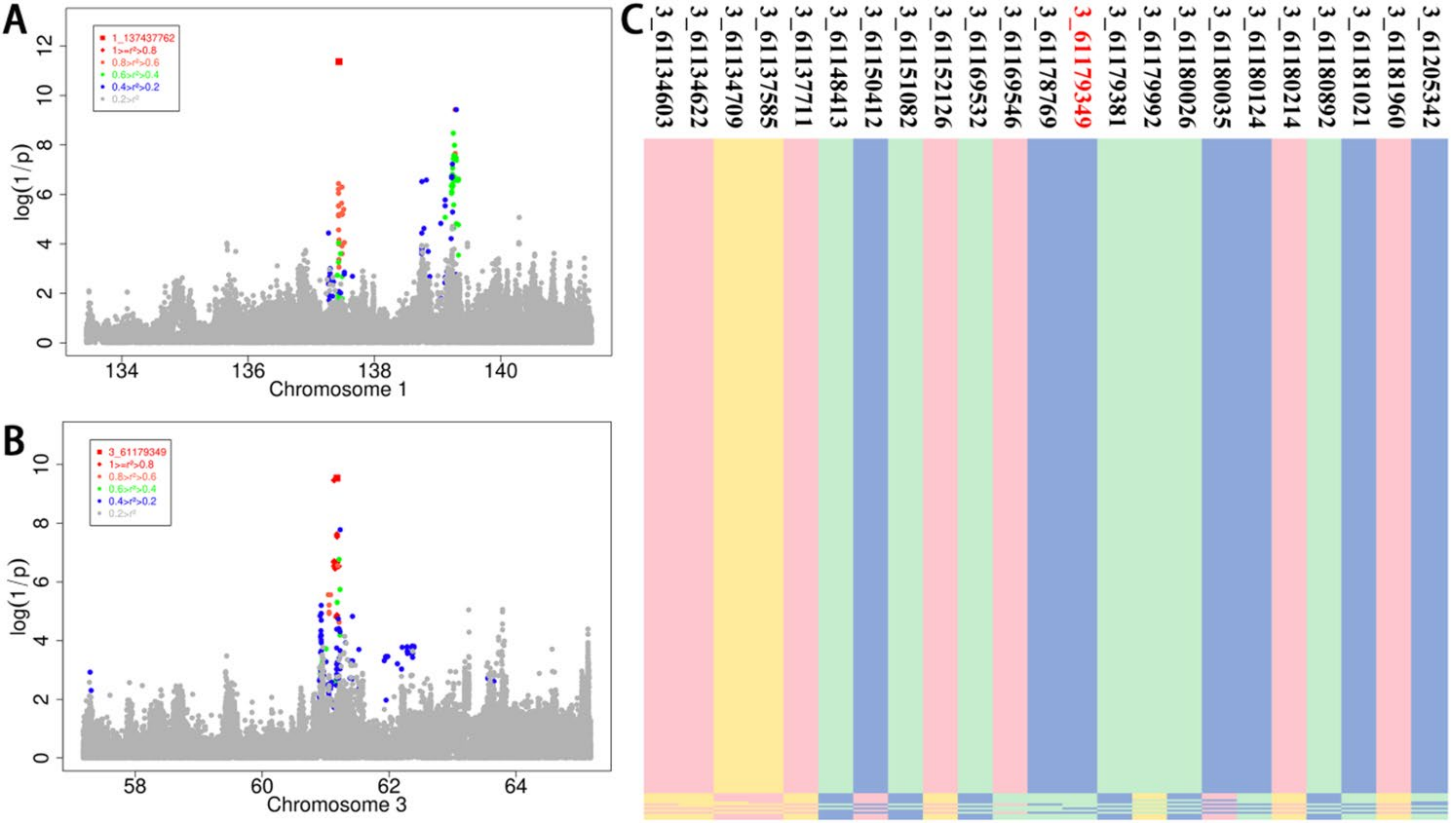
Regional plots of GWAS signals for LMW in chromosome 1 (**A**) and chromosome 3 (**B**). The most significant locus is indicated by square red dots, and the color of the other loci represents the linkage coefficient between the most significant locus and other loci (red highest). **C** was the haplotype sharing within the confidence interval for LMW in chromosome 3. Pink, yellow, green, and blue represent genotypes G, A, C, and T, respectively. The top displays SNP information, indicated as chromosome_position

LD analysis was conducted on SNPs located within significant *QTLs* on chromosomes 1 and 3, which were selected for their genome-wide association with LMW traits. The most significant association was observed with *SNP* 1_137437762, which was found to be in strong LD with 132 other *SNPs* within a 1.93 Mb region spanning from 137.40 Mb to 139.33 Mb on chromosome 1 (Fig. 4A, Additional file 1: Table S4). Similarly, SNP 3_61180214, one of 49 *SNPs* with substantial LD status within a 0.31 Mb region spanning from 60.91 Mb to 61.22 Mb on chromosome 3, showed the strongest association (Fig. 3B, Additional file 1: Table S4).

We performed a haplotype sharing analysis on chromosome 3, focusing on loci with LD greater than 0.8. This analysis identified 320 sequences sharing a specific haplotype, termed the “Shared-haplotype,” with a mean phenotype (LMW? ) value of 95.19. The remaining 12 sequences, classified as “Chaotic-haplotype,” had a mean phenotype value of 86.75 (Fig. 4C, Additional file 2: Table S1). A t-test comparing these groups revealed that the Shared-haplotype significantly influences LMW (*p*-value = 0.0047).

### Bootstrap test

Our study acknowledges several limitations, notably the small sample size of our experimental population. To assess the robustness of our findings, we performed a bootstrap analysis examining the association between chromosome 1 and the LMW phenotype. Among 1,000 genome-wide association studies (GWAS), over 950 did not identify significant signals on chromosome 1 (P_wald < 7.41E-09). This finding suggests that the fluctuation in the data structure of our experimental population did not affect the GWAS (FDR < 0.05).

## Discussion

Understanding the genetic basis of growth and carcass traits is essential for enhancing meat yield and quality in duck production, thereby improving profitability and sustainability. While the Shaoxing duck is recognized as a valuable genetic resource, its phenotypic and genetic characteristics remain inadequately studied. In this study, we evaluated its phenotypic and genetic parameters. In general, this breed is relatively small in body size and has a slow growth rate, which is possibly helpful for balancing body weight and bone development [28].

In animal breeding, accurately estimating genetic parameters is essential for effective selection and improvement. Pedigree errors are common in commercial breeding, leading to reduced accuracy in genetic evaluations [29]. Utilizing a genomic relationship matrix derived from high-density genotyping can effectively correct these errors, enhancing the precision of genetic parameter estimates [30]. Our study on Shaoxing ducks found that body size and carcass traits exhibited low to moderate heritability. These estimates are generally lower compared to those reported for Beijing ducks and Sichuan shelducks. This variation may be attributed to differences in populations and estimation methodologies [9, 31]. The relatively small number of experimental individuals used in this study is also one of the reasons for the ‘loss’ of heritability. In poultry breeding, The measurement of carcass traits is both expensive and difficult, and can only be conducted after death. In contrast, Slaughter Weight (SW) is an important live measurement that shows a very high phenotypic correlation with Eviscerated Weight (EW), Leg Muscle Weight (LMW), and Half-eviscerated Weight (HEW). This suggests that it is possible to improve two important carcass traits, LMW and HEW, without slaughter measurements by selecting for SW, or to directly enhance the carcass performance of Shaoxing ducks through selection based on SW. Our findings provide valuable insights for the breeding of carcass traits in Shaoxing ducks.

In this study, the *RSPO3* gene and *SPATA13* gene are significantly associated with BMW and LMW. According to the *QTL* information of ducks, the *RSPO3* (R-spondin3) gene is one of the members of the R-spondin family, primarily involved in the regulation of the Wnt/β-catenin signaling pathway [32]. Related studies have shown that this pathway plays a key role in embryonic development, muscle growth, and bone formation. Previous studied indicated that the *RSPO3* gene acts as an enhancer of Wnt signaling and may influence the breast muscle weight of broiler chickens [33]. In studies related to some mammals (such as humans and mice), *SPATA13* has been shown to play an important role in regulating cell migration, cell adhesion, and signal transduction. This suggests that *SPATA13* may influence growth and development, as well as body composition, through mechanisms that regulate the proliferation and differentiation of muscle cells or the remodeling of the extracellular matrix [34].

**Fig. 5:**
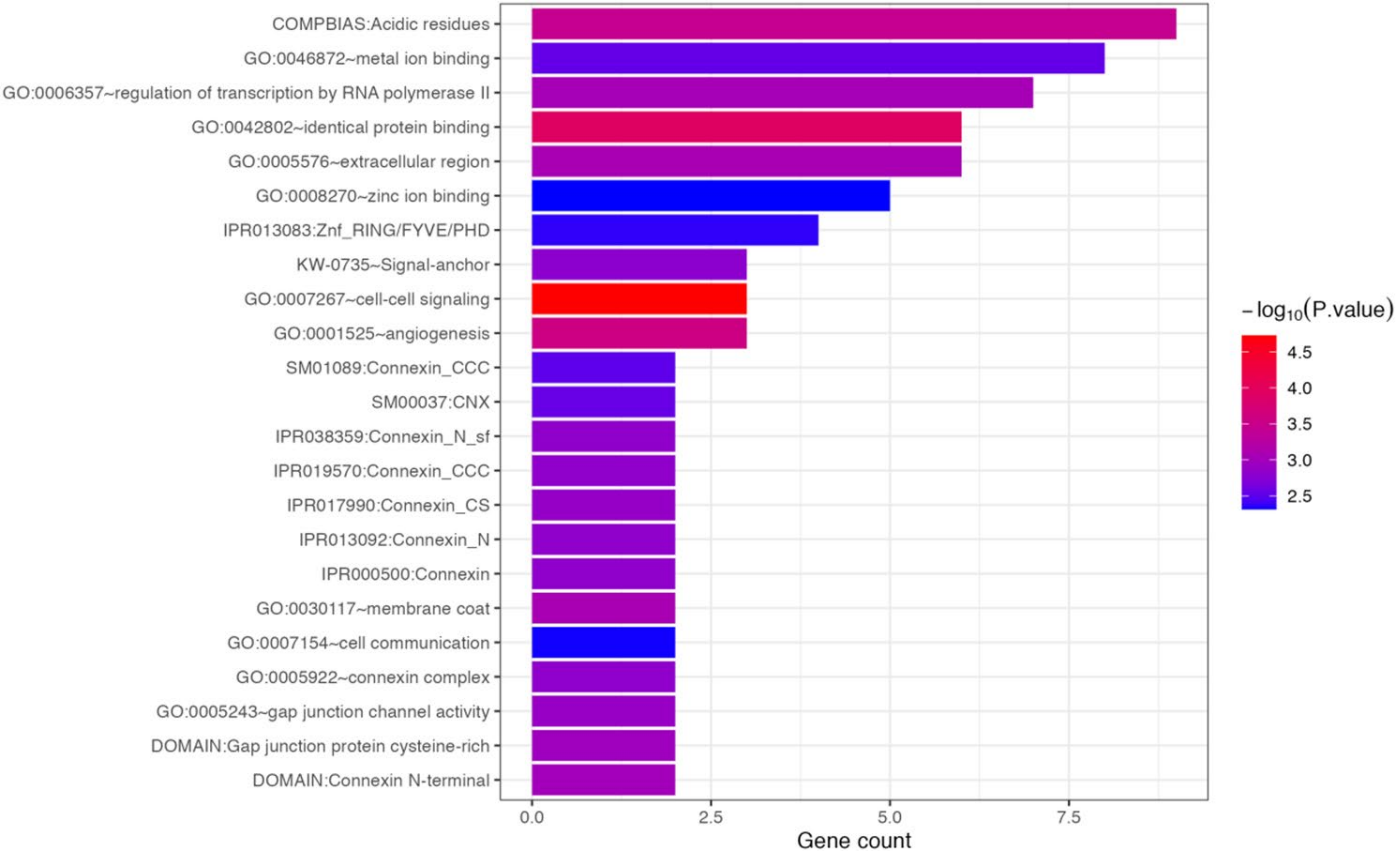
Gene GO enrichment results chart, with the horizontal axis representing the counts of genes and the vertical axis representing the enriched terms

Through literature search, the Collagen type VI Alpha 3 chain gene (*COL6A3*) being significantly associated with EWP and HEWP. The *COL6A3* produces collagen molecules found in the extracellular matrix and surrounding cells that make up the muscles used for movement. This indicates that *COL6A3* may be related to muscle cell integrity and the structure and composition of muscle fibers [35]. Among the candidate genes related to LMW that we focused on, *FGF9*, *LATS2*, and *PFKP* are involved in muscle or energy metabolism regulation, suggesting their potential relevance to poultry muscle development and warranting further attention [36]. We performed GO analysis on all candidate genes using the DAVID website, and identified 23 significant pathways (Fig. 5, Additional file 2: Table S2). The most significant pathway was GO:0007267 (cell–cell signaling), which is a broad GO term that essentially encompasses multiple specific pathways (such as Notch, Wnt/β-catenin, Hedgehog/Ihh, IGF/PI3K-AKT, TGF-β, etc.). These specific pathways have been extensively studied and confirmed to be related to skeletal growth (proliferation/hypertrophy of growth plate chondrocytes), myogenesis and satellite cell regulation, as well as muscle fiber growth and differentiation.

In addition, we identified a haplotype on chromosome 3 that is highly significantly related to the LMW trait, which provides valuable insights and guidance for future breeding programs of LMW. However, the relatively small sample size of our experimental population is a limitation of this study. To address this, we employed a bootstrap test to verify the reliability of our GWAS results. The test confirmed that the significant signals obtained in our GWA study were not due to chance, and therefore can be considered reliable. The method we used is available on Github at the following address: https://github.com/xuwenwu248/bootstrap.sh/commit/07db59d129c24c384ba2c43f9bb3f8d62fce845b.

## Conclusion

This study presents a GWAS analysis of body size and carcass yields traits in Shaoxing ducks, identifying a total of 50 candidate genes. LD analysis and haplotype sharing analysis revealed one haplotype strongly linked with LMW. This is the first GWAS publication on body size and carcass yields in Shaoxing ducks, contributing to the limited knowledge on the levels and heritability of these components. Our findings suggest potential biomarkers that could be utilized in breeding programs for duck-related traits.

## Supplementary Information

Supplementary Material 1.

Supplementary Material 2.

## Data Availability

The raw sequence data reported in this paper have been deposited in the Genome Sequence Archive in National Genomics Data Center, China National Center for Bioinformation / Beijing Institute of Genomics, Chinese Academy of Sciences (GSA: CRA004587) that are publicly accessible at https://ngdc.cncb.ac.cn/gsa.

## References

[1] Magdelaine P, Spiess M, Valceschini EJWPSJ. Poultry meat consumption trends in Europe. World's Poult Sci J. 2008;64(1):53–64.

[2] Tai C, Tai J-JLJTJPS. Future prospects of duck production in Asia. J Poult Sci. 2001;38(1):99–112.

[3] Whitton C, Bogueva D, Marinova D, Phillips CJJA. Are we approaching peak meat consumption? Analysis of meat consumption from 2000 to 2019 in 35 countries and its relationship to gross domestic product. Animals. 2021;11(12):3466. 10.3390/ani11123466 PMC8697883 34944243

[4] Liu H, Wang L, Guo Z, Xu Q, Fan W, Xu Y, Hu J, Zhang Y, Tang J, Xie MJGSE. Genome-wide association and selective sweep analyses reveal genetic loci for FCR of egg production traits in ducks. Genet Sel Evol. 2021;53:1–16. 10.1186/s12711-021-00684-5 PMC8690979 34930109

[5] Klein RJ, Zeiss C, Chew EY, Tsai JY, Sackler RS, Haynes C, Henning AK, SanGiovanni JP, Mane SM, Mayne ST, et al. Complement factor H polymorphism in age-related macular degeneration. Science. 2005;308(5720):385–9. 15761122 10.1126/science.1109557 PMC1512523

[6] Li Y-d, Xue B, Xin L, Wang W-j, Li Z-w, Ning W, Fan X, Gao H-h. Guo H-s, Hui ljjoia: integration of genome-wide association study and selection signatures reveals genetic determinants for skeletal muscle production traits in an F2 chicken population. Journal of Integrative Agriculture. 2022;21(7):2065–75.

[7] Ding J, Ying F, Li Q, Zhang G, Zhang J, Liu R, Zheng M, Wen J, Zhao GJJoAS. Biotechnology: A significant quantitative trait locus on chromosome Z and its impact on egg production traits in seven maternal lines of meat-type chicken. J Anim Sci Biotech. 2022;13(1):96. 10.1186/s40104-022-00744-w PMC9361671 35941697

[8] Li X, Xin A, Ma L, Gou X, Fang S, Dong X, Ni B, Tang L, Zhu L. Yan djfivs: molecular genetic characterization and meat-use functional gene identification in Jianshui yellow–brown ducks through combined resequencing and transcriptome analysis. Front Vet Sci. 2023;10:1269904. 10.3389/fvets.2023.1269904 PMC10765987 38179331

[9] Deng MT, Zhu F, Yang YZ, Yang FX, Hao JP, Chen SR, Hou ZC. Genome-wide association study reveals novel loci associated with body size and carcass yields in Pekin ducks. BMC Genomics. 2019;20(1):1. 30606130 10.1186/s12864-018-5379-1 PMC6318962

[10] Zhou Z, Li M, Cheng H, Fan W, Yuan Z, Gao Q, Xu Y, Guo Z, Zhang Y, Hu J, et al. An intercross population study reveals genes associated with body size and plumage color in ducks. Nat Commun. 2018;9(1):2648. 30018292 10.1038/s41467-018-04868-4 PMC6050300

[11] Li L, Quan J, Gao C, Liu H, Yu H, Chen H, Xia C, Zhao SJPS. Whole-genome resequencing to unveil genetic characteristics and selection signatures of specific pathogen-free ducks. Poult Sci. 2023;102(7):102748. 10.1016/j.psj.2023.102748 PMC10208885 37209656

[12] Zhu T, Qi X, Chen Y, Wang L, Lv X, Yang W, Zhang J, Li K, Ning Z, Jiang ZJBE et al. Positive selection of skeleton-related genes during Duck domestication revealed by whole genome sequencing. BMC Ecol Evol. 2021;21:1–8. 10.1186/s12862-021-01894-7 PMC8419914 34488647

[13] Deng M-T, Zhu F, Yang Y-Z, Yang F-X, Hao J-p, Chen S-R, Hou Z-CJBG. Genome-wide association study reveals novel loci associated with body size and carcass yields in Pekin ducks. BMC Genomics. 2019;20:1–13. 10.1186/s12864-018-5379-1 PMC6318962 30606130

[14] Deng MT, Zhang F, Zhu F, Yang YZ, Yang FX, Hao JP, Hou ZCJAG. Genome-wide association study reveals novel loci associated with fat‐deposition and meat‐quality traits in Pekin ducks. Anim Genet. 2020;51(6):953–7. 10.1111/age.12995 32844456

[15] Liang S, Guo Z, Luo D, Tang J, Ji Z, Xie M, Hou SJG. Two variants of AUTS2 gene are associated with high lean meat percentage in Pekin ducks. Gene. 2023;848:146864. 10.1016/j.gene.2022.146864 36067863

[16] Hu Z-L, Park CA, Reecy JMJN. Developmental progress and current status of the animal QTLdb. Nucleic Acids Res. 2016;44(D1):D827–33. 10.1093/nar/gkv1233 PMC4702873 26602686

[17] Lin F-B, Zhu F, Hao J-P, Yang F-X, Hou Z-CJPS. In vivo prediction of the carcass fatness using live body measurements in Pekin ducks. Poult Sci. 2018;97(7):2365–71. 10.3382/ps/pey079 29618042

[18] McCaw ZR, Lane JM, Saxena R, Redline S, Lin X. Operating characteristics of the rank-based inverse normal transformation for quantitative trait analysis in genome-wide association studies. Biometrics. 2020;76(4):1262–72. 31883270 10.1111/biom.13214 PMC8643141

[19] Jiang Y, Jiang Y, Wang S, Zhang Q, Ding X. Optimal sequencing depth design for whole genome re-sequencing in pigs. BMC Bioinformatics. 2019;20(1):556. 31703550 10.1186/s12859-019-3164-z PMC6839175

[20] Li H, Durbin R. Fast and accurate short read alignment with Burrows-Wheeler transform. Bioinformatics. 2009;25(14):1754–60. 19451168 10.1093/bioinformatics/btp324 PMC2705234

[21] McKenna A, Hanna M, Banks E, Sivachenko A, Cibulskis K, Kernytsky A, Garimella K, Altshuler D, Gabriel S, Daly M, et al. The genome analysis toolkit: a mapreduce framework for analyzing next-generation DNA sequencing data. Genome Res. 2010;20(9):1297–303. 20644199 10.1101/gr.107524.110 PMC2928508

[22] Cingolani P, Platts A, Wang le L, Coon M, Nguyen T, Wang L, Land SJ, Lu X, Ruden DM. A program for annotating and predicting the effects of single nucleotide polymorphisms, snpeff: SNPs in the genome of drosophila melanogaster strain w1118; iso-2; iso-3. Fly (Austin). 2012;6(2):80–92. 22728672 10.4161/fly.19695 PMC3679285

[23] Zhou X, Stephens M. Genome-wide efficient mixed-model analysis for association studies. Nat Genet. 2012;44(7):821–4. 22706312 10.1038/ng.2310 PMC3386377

[24] Yang Q, Cui J, Chazaro I, Cupples LA, Demissie S. Power and type I error rate of false discovery rate approaches in genome-wide association studies. BMC Genet. 2005;6(Suppl 1):S134. 16451593 10.1186/1471-2156-6-S1-S134 PMC1866802

[25] Pearson TA, Manolio TA. How to interpret a genome-wide association study. JAMA. 2008;299(11):1335–44. 18349094 10.1001/jama.299.11.1335

[26] Chang CC, Chow CC, Tellier LC, Vattikuti S, Purcell SM, Lee JJ. Second-generation PLINK: rising to the challenge of larger and richer datasets. Gigascience. 2015;4:7. 25722852 10.1186/s13742-015-0047-8 PMC4342193

[27] Scheet P, Stephens M. A fast and flexible statistical model for large-scale population genotype data: applications to inferring missing genotypes and haplotypic phase. Am J Hum Genet. 2006;78(4):629–44. 16532393 10.1086/502802 PMC1424677

[28] Zhang HY, Zeng QF, Bai SP, Wang JP, Ding XM, Xuan Y, Su ZW, Fraley GS, Zhang KY. Study on the morphology and mineralization of the tibia in meat ducks from 1 to 56 d. Poult Sci. 2019;98(9):3355–64. 30916353 10.3382/ps/pez121

[29] Zhu F, Cheng SR, Yang YZ, Hao JP, Yang FX, Hou ZC. Genome-Wide association study of growth and feeding traits in Pekin ducks. Front Genet. 2019;10:702. 31404312 10.3389/fgene.2019.00702 PMC6676418

[30] Goddard ME, Hayes BJ, Meuwissen TH. Using the genomic relationship matrix to predict the accuracy of genomic selection. J Anim Breed Genet. 2011;128(6):409–21. 22059574 10.1111/j.1439-0388.2011.00964.x

[31] Yang Z, Xi Y, Qi J, Li L, Bai L, Zhang J, Lv J, Li B, Liu H. Genome-wide association studies reveal the genetic basis of growth and carcass traits in Sichuan Shelduck. Poult Sci. 2024;103(11):104211. 39216264 10.1016/j.psj.2024.104211 PMC11402601

[32] Alhazmi N, Carroll SH, Kawasaki K, Woronowicz KC, Hallett SA, Macias Trevino C, Li EB, Baron R, Gori F, Yelick PC, et al. Synergistic roles of Wnt modulators R-spondin2 and R-spondin3 in craniofacial morphogenesis and dental development. Sci Rep. 2021;11(1):5871. 33712657 10.1038/s41598-021-85415-y PMC7954795

[33] Dou D, Shen L, Zhou J, Cao Z, Luan P, Li Y, Xiao F, Guo H, Li H, Zhang H. Genome-wide association studies for growth traits in broilers. BMC Genom Data. 2022;23(1):1. 34979907 10.1186/s12863-021-01017-7 PMC8725492

[34] Szabo L, Morey R, Palpant NJ, Wang PL, Afari N, Jiang C, Parast MM, Murry CE, Laurent LC, Salzman J. Statistically based splicing detection reveals neural enrichment and tissue-specific induction of circular RNA during human fetal development. Genome Biol. 2015;16(1):126. 26076956 10.1186/s13059-015-0690-5 PMC4506483

[35] Pampouille E, Berri C, Boitard S, Hennequet-Antier C, Beauclercq SA, Godet E, Praud C, Jego Y, Le Bihan-Duval E. Mapping QTL for white striping in relation to breast muscle yield and meat quality traits in broiler chickens. BMC Genomics. 2018;19(1):202. 29554873 10.1186/s12864-018-4598-9 PMC5859760

[36] Zlibut A, Bocsan IC, Agoston-Coldea L. Pentraxin-3 and endothelial dysfunction. Adv Clin Chem. 2019;91:163–79. 31331488 10.1016/bs.acc.2019.03.005

