# Supplementary material for: Genome-wide association study reveals novel loci associated with body size and carcass yields in Shaoxing ducks
Source: BMC Genomics. 2025 Dec 9;27:37. doi: 10.1186/s12864-025-12411-1 (PMC12802297; doi:10.1186/s12864-025-12411-1)
Supplement: Supplementary file 2 — Supplementary Material 2. [file 12864_2025_12411_MOESM2_ESM.docx]

# Supplementary materials for

**Genome-wide association study reveals novel loci associated with body size and carcass yields in Shaoxing ducks**

Wenwu Xu^1+*^, Zhaobin Wang^1+^, Tao Zeng^1^, Yong Tian^1^, Tiantian Gu^1^, Li Chen^1^, Lizhi Lu^1^

*Correspondence Author: Wenwu Xu: xuwenwu248@outlook.com; Lizhi Lu: [lulizhibox@163.com](mailto:lulizhibox@163.com)


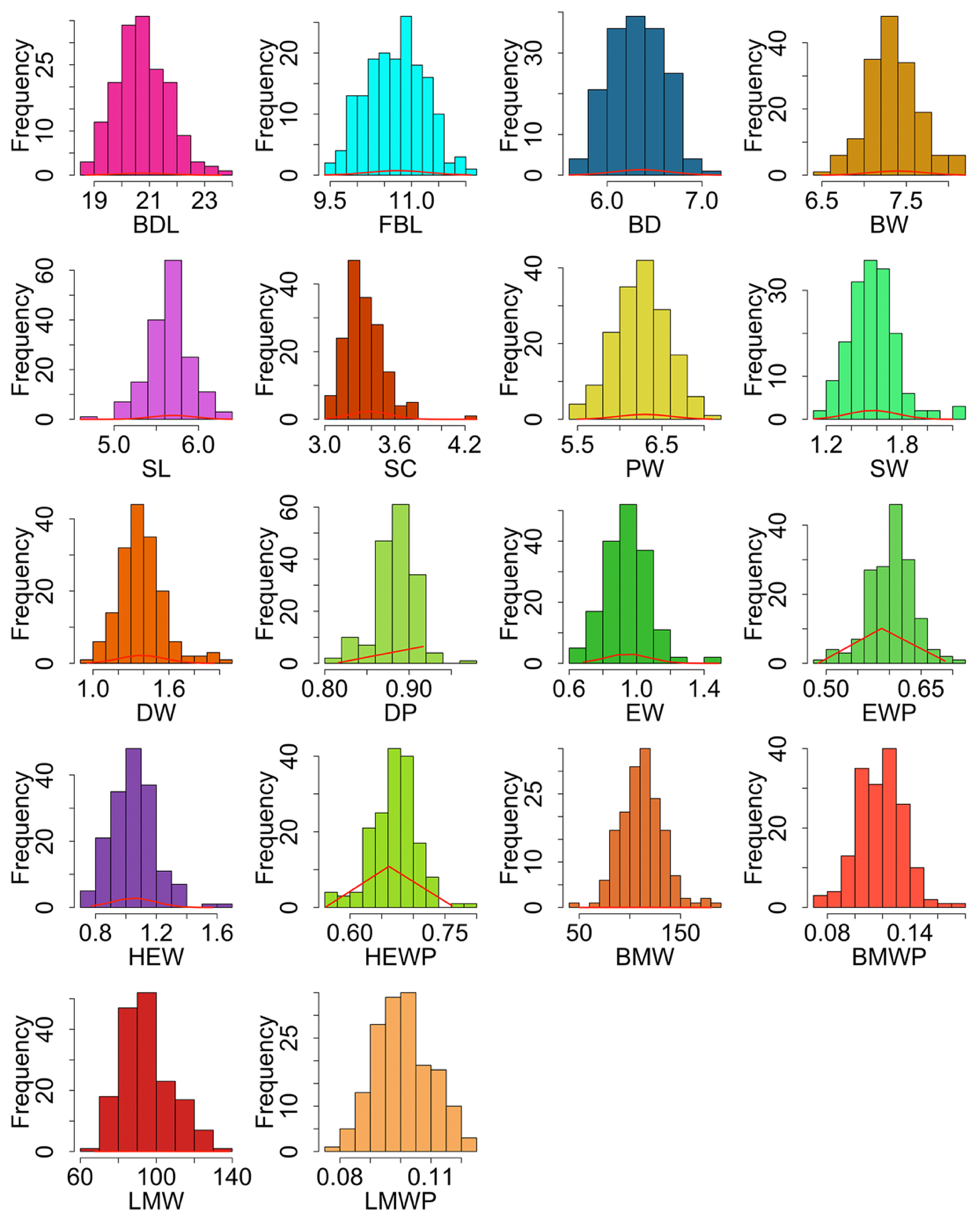


**Figure S1 |** Histogram distribution of the eighteen phenotypes. The horizontal axis represents the corresponding phenotype, the vertical axis denotes frequency, and the red solid line is the fitted curve of the normal distribution.

**
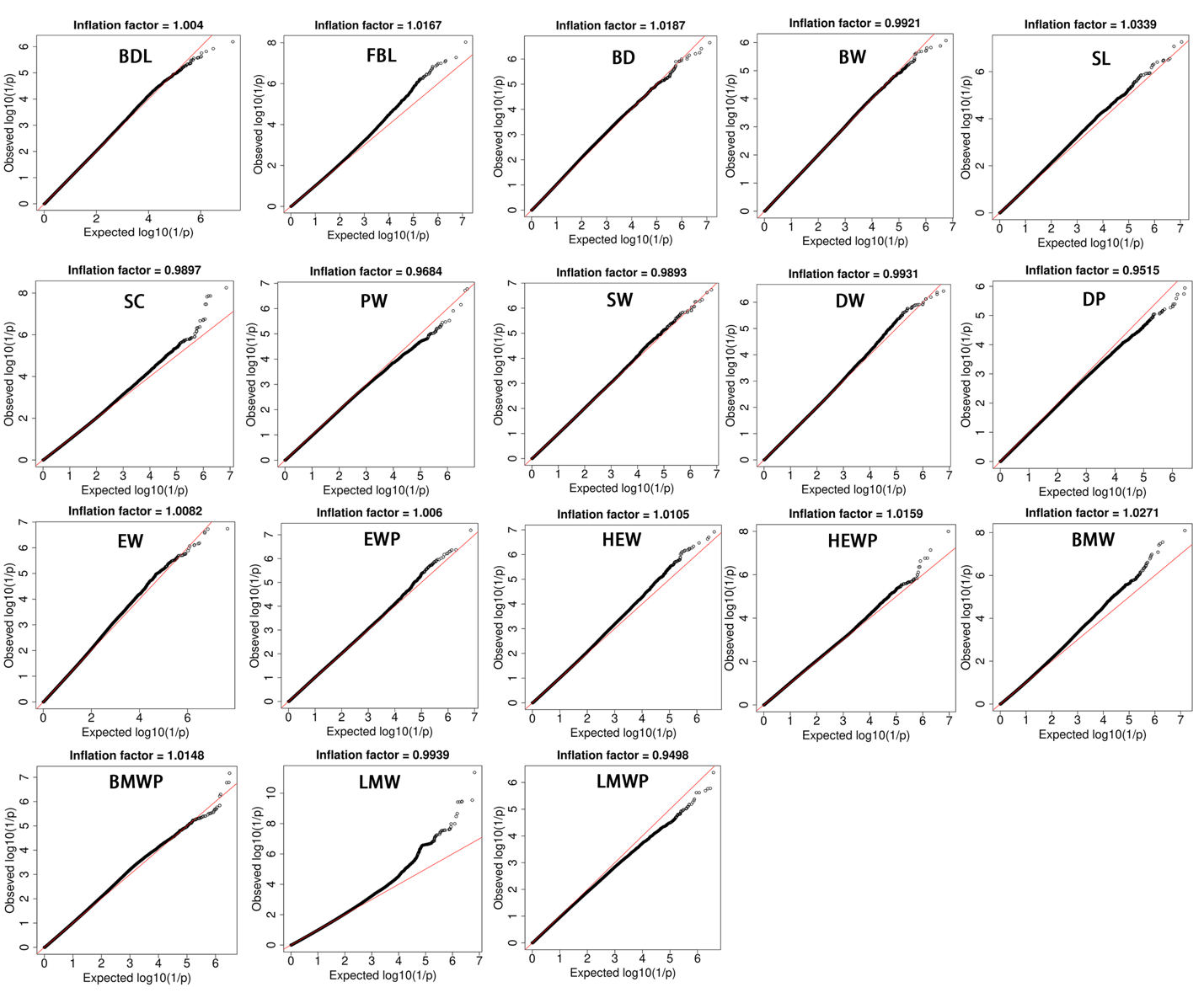
**

**Figure S2 |** Q-Q plots derived from GWASs for BDL, FBL, BD, BW,SL, SC, PW, SW, DW, DP, EW, EWP, HEW, HEWP, BMW, BMWP, LMW, and LMWP. The Q-Q plot contains expected -log10-transformed P-values plotted against observed -log10-transformed P-values. Inflation factor denotes the genomic inflation factor indicating the degree of population stratification.

**
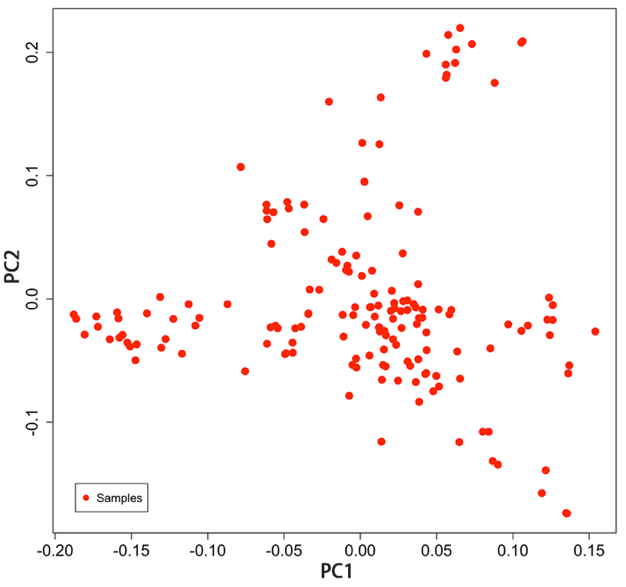
**

**Figure S3 |**The results of PCA. PC1 and PC2 represent two principal components, and the red dots in the figure represent the experimental subjects.

**Table S1** The Sequencing quality for 166 Shaoxing ducks.

|  | Sequencing quality | | | | | |
| --- | --- | --- | --- | --- | --- | --- |
| ID | Raw_Reads | Clean_Reads | Clean_Base | Q20(%) | Q30(%) | GC(%) |
| R01 | 45146828 | 45062628 | 13500079976 | 96.63 | 91.85 | 42.45 |
| R02 | 47806709 | 47739891 | 14302494876 | 96.76 | 92.11 | 42.43 |
| R03 | 39834024 | 39792267 | 11921666508 | 96.41 | 91.3 | 41.55 |
| R04 | 46180811 | 46120792 | 13817568926 | 96.35 | 91.18 | 42.16 |
| R05 | 40008462 | 39958494 | 11970966510 | 96.26 | 91.05 | 42.09 |
| R06 | 43491075 | 43400535 | 13000201476 | 96.68 | 91.93 | 42.14 |
| R07 | 46505176 | 46407426 | 13902379082 | 96.78 | 92.1 | 42.54 |
| R08 | 44912092 | 44848487 | 13436130334 | 96.85 | 92.26 | 42.27 |
| R09 | 44649055 | 44602106 | 13361767268 | 96.72 | 91.97 | 41.82 |
| R10 | 49772929 | 49694665 | 14887643538 | 96.07 | 90.72 | 42.35 |
| R11 | 50699628 | 50628198 | 15167841974 | 96.74 | 92.07 | 42.29 |
| R12 | 53688184 | 53612346 | 16061897954 | 96.36 | 91.3 | 42.27 |
| R13 | 52675713 | 52603634 | 15759996228 | 96.6 | 91.76 | 42.44 |
| R14 | 53165108 | 53090635 | 15905169708 | 96.63 | 91.86 | 42.23 |
| R15 | 51001741 | 50932508 | 15258783756 | 96.51 | 91.6 | 42.04 |
| R16 | 53008486 | 52933832 | 15858373986 | 96.5 | 91.57 | 41.97 |
| R17 | 44776953 | 44708252 | 13394115692 | 96.16 | 90.87 | 42.43 |
| R18 | 53599020 | 53531148 | 16037818118 | 96.68 | 91.77 | 42.21 |
| R19 | 61823045 | 61740222 | 18497193582 | 96.79 | 92.1 | 41.78 |
| R20 | 50202520 | 50128770 | 15018190608 | 96.36 | 91.29 | 42.03 |
| R21 | 54969789 | 54900476 | 16447713748 | 96.49 | 91.49 | 41.85 |
| R22 | 55688538 | 55601933 | 16657553088 | 96.29 | 91.16 | 42.24 |
| R23 | 52741275 | 52647558 | 15772038438 | 96.81 | 92.22 | 42.02 |
| R24 | 52382936 | 52299785 | 15668412920 | 96.23 | 91 | 41.82 |
| R25 | 55050145 | 54940300 | 16457546388 | 96.52 | 91.57 | 41.84 |
| R26 | 55818222 | 55745647 | 16701172444 | 96.79 | 92.02 | 42.02 |
| R27 | 50257609 | 50174555 | 15031661246 | 96.63 | 91.9 | 42.09 |
| R28 | 40675851 | 40604298 | 12163984160 | 96.65 | 91.87 | 42.35 |
| R29 | 39430165 | 39369761 | 11794304838 | 96.66 | 91.81 | 42.04 |
| R30 | 41017162 | 40945838 | 12266026766 | 97 | 92.52 | 41.6 |
| R31 | 48193394 | 48127582 | 14418670078 | 96.83 | 92.2 | 41.88 |
| R32 | 44662172 | 44601003 | 13362118916 | 96.8 | 92.21 | 42.54 |
| R33 | 60628937 | 60540379 | 18137327768 | 96.59 | 91.73 | 41.98 |
| R34 | 50142367 | 50070451 | 15000740510 | 96.74 | 92.07 | 42.37 |
| R35 | 42419171 | 42329838 | 12680131754 | 96.03 | 90.49 | 41.95 |
| R36 | 46361057 | 46305054 | 13872431924 | 96.85 | 92.28 | 42.05 |
| R37 | 42552909 | 42464322 | 12720530566 | 96.47 | 91.43 | 41.8 |
| R38 | 44661780 | 44590110 | 13358490650 | 96.69 | 91.92 | 41.95 |
| R39 | 47700284 | 47642092 | 14272966788 | 96.72 | 92 | 42.05 |
| R40 | 50264963 | 50192206 | 15036613216 | 95.91 | 90.01 | 42.11 |
| R41 | 47390924 | 47318838 | 14176017396 | 96.56 | 91.65 | 41.95 |
| R42 | 46932795 | 46862356 | 14039144190 | 96.71 | 92.04 | 42.05 |
| R43 | 49426030 | 49354294 | 14786437404 | 96.56 | 91.72 | 42.36 |
| R44 | 48081809 | 48025062 | 14388563564 | 96.69 | 91.83 | 42.18 |
| R45 | 54281461 | 54216218 | 16242002176 | 96.83 | 92.17 | 42.08 |
| R46 | 40903748 | 40850840 | 12238872038 | 96.78 | 92.01 | 42.38 |
| R47 | 49935720 | 49872983 | 14941554618 | 96.06 | 90.54 | 42.37 |
| R48 | 61037511 | 60956052 | 18261706404 | 96.47 | 91.53 | 42.46 |
| R49 | 50029650 | 49987930 | 14976276134 | 97.01 | 92.4 | 42.24 |
| R50 | 62738515 | 62640558 | 18766225392 | 96.76 | 92.1 | 42.36 |
| R51 | 43701222 | 43649450 | 13077169442 | 96.75 | 92.08 | 42.31 |
| R52 | 46384795 | 46327130 | 13879388024 | 96.53 | 91.55 | 42.31 |
| R53 | 44285285 | 44223900 | 13249048766 | 96.78 | 92.16 | 42.38 |
| R54 | 42485568 | 42432958 | 12712303378 | 96.66 | 91.93 | 42.48 |
| R55 | 37240380 | 37181028 | 11139044796 | 96.09 | 90.75 | 42.48 |
| R56 | 47657142 | 47584512 | 14255890786 | 96.6 | 91.78 | 42.42 |
| R57 | 48872628 | 48802887 | 14621108216 | 96.63 | 91.86 | 42.39 |
| R58 | 59800333 | 59705520 | 17886972370 | 96.51 | 91.61 | 42.43 |
| R59 | 43928273 | 43894039 | 13151522718 | 96.88 | 92.17 | 42.17 |
| R60 | 57434091 | 57342210 | 17178773302 | 96.64 | 91.89 | 42.32 |
| R61 | 44681368 | 44633811 | 13372186494 | 96.81 | 92.16 | 42.35 |
| R62 | 49961056 | 49887501 | 14945528392 | 96.15 | 90.86 | 42.3 |
| R63 | 50553747 | 50481934 | 15123805506 | 96.25 | 91.03 | 42.17 |
| R64 | 36794962 | 36748080 | 11009497418 | 96.71 | 92.02 | 42.4 |
| R65 | 47037008 | 46973347 | 14072875768 | 96.34 | 91.2 | 42.11 |
| R66 | 45872937 | 45813106 | 13725397610 | 96.39 | 91.3 | 42.29 |
| R67 | 57036806 | 56956040 | 17063935364 | 96.47 | 91.54 | 42.52 |
| R68 | 51545117 | 51461460 | 15417254588 | 96.59 | 91.79 | 42.36 |
| R69 | 43139061 | 43064424 | 12901461846 | 96.46 | 91.53 | 42.36 |
| R70 | 41277476 | 41214996 | 12347427260 | 96.18 | 90.95 | 42.48 |
| R71 | 37243558 | 37193593 | 11143103598 | 96.65 | 91.87 | 42.52 |
| R72 | 43143039 | 43060171 | 12899006438 | 96.64 | 91.85 | 42.26 |
| R73 | 38126453 | 38072349 | 11405816130 | 96.59 | 91.76 | 41.84 |
| R74 | 46377175 | 46326131 | 13878644688 | 96.32 | 91.18 | 42.34 |
| R75 | 43199483 | 43134958 | 12921575252 | 97.29 | 93.22 | 42.54 |
| R76 | 52414350 | 52352469 | 15684200506 | 96.67 | 91.88 | 42.16 |
| R77 | 44872498 | 44807170 | 13423370328 | 96.46 | 91.49 | 42.34 |
| R78 | 69010754 | 68837814 | 20620779724 | 96.13 | 90.8 | 43.9 |
| R79 | 65505548 | 65350473 | 19576049474 | 95.57 | 89.62 | 43.85 |
| R80 | 62816481 | 62712608 | 18787671008 | 96.26 | 90.92 | 43.26 |
| R81 | 74313394 | 74192206 | 22226305210 | 96.6 | 91.67 | 43.29 |
| R82 | 50454085 | 50379537 | 15093196308 | 96.28 | 90.93 | 43.64 |
| R83 | 58961420 | 58855228 | 17632017236 | 96.46 | 91.48 | 44.22 |
| R84 | 52269340 | 52198887 | 15638317002 | 96.34 | 91.1 | 43.63 |
| R85 | 52772827 | 52677049 | 15780932618 | 96.29 | 91.03 | 43.72 |
| R86 | 68963713 | 68835420 | 20621200508 | 96.48 | 91.44 | 43.74 |
| R87 | 87018223 | 86739077 | 25979195624 | 96.06 | 90.96 | 44.65 |
| R88 | 78580438 | 78452394 | 23503348466 | 96.43 | 91.26 | 43.75 |
| R89 | 59633626 | 59514981 | 17828977750 | 96.44 | 91.36 | 44.07 |
| R90 | 63363040 | 63262332 | 18952177512 | 96.45 | 91.33 | 43.74 |
| R91 | 55417475 | 55289139 | 16562894642 | 96.04 | 90.52 | 43.88 |
| R92 | 44048035 | 43954282 | 13167514972 | 96.32 | 91.05 | 43.83 |
| R93 | 57803446 | 57688269 | 17281579970 | 96.38 | 91.2 | 43.65 |
| R94 | 79225140 | 79093674 | 23695345478 | 96.6 | 91.69 | 43.81 |
| R95 | 42635557 | 42581333 | 12757625156 | 96.62 | 91.71 | 43.87 |
| R96 | 65749413 | 65630677 | 19662084990 | 96.08 | 90.66 | 44.5 |
| R97 | 42811001 | 42531271 | 12741920106 | 96.42 | 91.39 | 43.76 |
| R98 | 40289017 | 39963485 | 11971985164 | 96.38 | 91.34 | 43.84 |
| R99 | 51351658 | 51286508 | 15365547634 | 96.54 | 91.41 | 43.35 |
| R100 | 37947059 | 34728007 | 10403776882 | 96 | 90.42 | 43.36 |
| R101 | 41365113 | 37837068 | 11335115030 | 95.86 | 90.27 | 44.01 |
| R102 | 40456171 | 40369524 | 12094475190 | 96.34 | 91.11 | 43.51 |
| R103 | 52499561 | 52416622 | 15703737746 | 96 | 90.49 | 43.64 |
| R104 | 56398096 | 56304081 | 16867988446 | 96.09 | 90.69 | 43.68 |
| R105 | 58438601 | 58351534 | 17481491568 | 96.33 | 91.14 | 43.26 |
| R106 | 48758526 | 48674487 | 14582628720 | 96.31 | 91.2 | 44.1 |
| R107 | 41284138 | 41160985 | 12330554632 | 95.73 | 89.94 | 43.77 |
| R108 | 41155740 | 41089285 | 12310180374 | 96.45 | 91.44 | 43.87 |
| R109 | 49407479 | 49298058 | 14768480056 | 96.09 | 90.85 | 44.49 |
| R110 | 52751779 | 52677201 | 15782099850 | 96.45 | 91.41 | 43.77 |
| R111 | 43149334 | 43084034 | 12908029184 | 95.99 | 90.45 | 43.75 |
| R112 | 49035436 | 48962232 | 14669156508 | 96.4 | 91.33 | 43.47 |
| R113 | 43276001 | 43058959 | 12895863456 | 95.07 | 89.42 | 46.22 |
| R114 | 48576812 | 48489785 | 14525578462 | 96.37 | 91.35 | 43.88 |
| R115 | 40508505 | 40456954 | 12120542876 | 96.44 | 91.38 | 43.81 |
| R116 | 51408640 | 51320481 | 15374764994 | 96 | 90.45 | 43.19 |
| R117 | 46937742 | 46857067 | 14037519388 | 96.23 | 90.92 | 43.33 |
| R118 | 46475424 | 46389955 | 13860223494 | 97.97 | 94.68 | 43.07 |
| R119 | 39164183 | 39096411 | 11713148332 | 96.35 | 91.3 | 44.17 |
| R120 | 39869134 | 39791458 | 11921526480 | 95.94 | 90.64 | 45.85 |
| R121 | 40248565 | 40195754 | 12042815884 | 96.43 | 91.37 | 43.82 |
| R122 | 42934137 | 42876336 | 12845953002 | 96.41 | 91.32 | 43.87 |
| R123 | 46133048 | 46056497 | 13798260274 | 96.37 | 91.3 | 43.81 |
| R124 | 49166925 | 49080142 | 14703927162 | 96 | 90.42 | 43.57 |
| R125 | 50899856 | 50766182 | 15208415018 | 96.01 | 90.81 | 45.68 |
| R126 | 48141254 | 47971677 | 14368841798 | 95.39 | 89.94 | 47.14 |
| R127 | 48106969 | 47940123 | 14303504184 | 97.79 | 94.25 | 43.16 |
| R128 | 43539492 | 43470685 | 13022717202 | 96.43 | 91.41 | 43.67 |
| R129 | 43576367 | 43492623 | 13028677336 | 96.09 | 90.72 | 43.43 |
| R130 | 48165794 | 48078749 | 14403283858 | 95.79 | 90.08 | 43.64 |
| R131 | 41811771 | 41734676 | 12502546760 | 96.38 | 91.34 | 43.58 |
| R132 | 50641432 | 50560178 | 15148162104 | 96 | 90.64 | 44.99 |
| R133 | 55025351 | 54937760 | 16458926840 | 95.98 | 90.5 | 43.5 |
| R134 | 48585171 | 48518133 | 14536376030 | 96.37 | 91.24 | 43.58 |
| R135 | 51939258 | 51810969 | 15482274406 | 97.93 | 94.69 | 43.04 |
| R136 | 57104921 | 57018080 | 17082873996 | 96.38 | 91.31 | 44.02 |
| R137 | 53967825 | 53867071 | 16137601152 | 96.2 | 90.92 | 43.6 |
| R138 | 49983381 | 49906525 | 14951876250 | 96.21 | 90.86 | 43.63 |
| R139 | 61386433 | 61262284 | 18353335458 | 96.29 | 91.11 | 43.62 |
| R140 | 46436818 | 46370752 | 13892907670 | 96.08 | 90.74 | 43.9 |
| R141 | 48376121 | 48283889 | 14464832796 | 96.11 | 90.77 | 43.64 |
| R142 | 48090354 | 48009810 | 14383117228 | 96.27 | 91.03 | 43.49 |
| R143 | 52146891 | 52071752 | 15600103636 | 96.2 | 91.02 | 43.92 |
| R144 | 43177450 | 43099956 | 12911894610 | 96.31 | 91.15 | 43.69 |
| R145 | 53173856 | 53088573 | 15904415400 | 96.39 | 91.31 | 43.56 |
| R146 | 42363894 | 42303380 | 12674080902 | 95.86 | 90.24 | 43.89 |
| R147 | 57293043 | 57209407 | 17139468684 | 96.24 | 90.97 | 43.48 |
| R148 | 55023060 | 54925376 | 16455608512 | 96.25 | 91.14 | 44.41 |
| R149 | 49692514 | 49629427 | 14869153628 | 96.23 | 90.95 | 43.7 |
| R150 | 53681467 | 53594062 | 16056213142 | 96.03 | 90.6 | 43.75 |
| R151 | 47891921 | 47797742 | 14320186388 | 96.08 | 90.77 | 44.68 |
| R152 | 45420476 | 45356298 | 13589154552 | 96.37 | 91.3 | 44.34 |
| R153 | 45409315 | 45316749 | 13577371310 | 96.24 | 90.87 | 43.59 |
| R154 | 38964641 | 38899364 | 11653935240 | 96.13 | 90.84 | 44.13 |
| R155 | 49539475 | 49446487 | 14813105974 | 96.31 | 91.23 | 43.8 |
| R156 | 53875659 | 53662063 | 16072166992 | 95.42 | 89.87 | 45.24 |
| R157 | 49997161 | 49910656 | 14952801210 | 96.48 | 91.48 | 43.56 |
| R158 | 58585313 | 58500940 | 17526153934 | 96.4 | 91.3 | 43.65 |
| R159 | 48544214 | 48478699 | 14523768568 | 96.28 | 91.1 | 43.6 |
| R160 | 54854551 | 54783592 | 16413352696 | 96.42 | 91.36 | 43.86 |
| R161 | 44593647 | 44513566 | 13334881512 | 96.25 | 91.13 | 43.78 |
| R162 | 44317165 | 44250843 | 13257385438 | 96.04 | 90.59 | 43.63 |
| R163 | 44193145 | 44112901 | 13215723388 | 95.91 | 90.4 | 43.72 |
| R164 | 51529215 | 51463062 | 15418614764 | 96.09 | 90.73 | 43.63 |
| R165 | 39795051 | 39688949 | 11890568484 | 95.93 | 90.55 | 45.22 |
| R166 | 46170226 | 46085754 | 13806660834 | 95.83 | 90.21 | 43.84 |

Raw_Reads represents the number of original reads, Clean_Reads represents the number of reads after quality control, Clean_Base represents the total number of bases after quality control, Q20(%) indicates the percentage of bases with a recognition accuracy above 99%, Q30(%) indicates the percentage of bases with a recognition accuracy above 99.9%, and GC(%) represents the percentage of G and C bases in the total bases.

Table S2 The Sequencing deqth for 166 Shaoxing ducks.

|  | Sequencing depth | | | |
| --- | --- | --- | --- | --- |
| ID | Ave_depth | Cov_ratio_1X(%) | Cov_ratio_5X(%) | Cov_ratio_10X(%) |
| R01 | 10 | 99.31 | 91.72 | 57.42 |
| R02 | 11 | 99.35 | 93.02 | 62.98 |
| R03 | 9 | 99.03 | 86.97 | 47.66 |
| R04 | 11 | 99.32 | 92.27 | 59.98 |
| R05 | 9 | 99.17 | 87.94 | 46.31 |
| R06 | 10 | 99.24 | 90.88 | 55.06 |
| R07 | 11 | 99.36 | 92.69 | 59.92 |
| R08 | 10 | 99.31 | 91.61 | 57.01 |
| R09 | 10 | 99.27 | 91.54 | 57.52 |
| R10 | 11 | 99.35 | 93.56 | 65.57 |
| R11 | 12 | 99.37 | 93.87 | 66.82 |
| R12 | 12 | 99.38 | 94.66 | 70.9 |
| R13 | 12 | 99.43 | 94.94 | 70.96 |
| R14 | 12 | 99.41 | 94.79 | 70.78 |
| R15 | 12 | 99.36 | 93.89 | 67.26 |
| R16 | 12 | 99.39 | 94.61 | 70.5 |
| R17 | 10 | 99.32 | 91.52 | 56.44 |
| R18 | 13 | 99.44 | 95.34 | 72.96 |
| R19 | 14 | 99.43 | 96.38 | 80.47 |
| R20 | 12 | 99.35 | 93.93 | 66.8 |
| R21 | 13 | 99.42 | 95.27 | 73.87 |
| R22 | 13 | 99.44 | 95.58 | 74.91 |
| R23 | 12 | 99.39 | 94.81 | 71.39 |
| R24 | 12 | 99.36 | 94.22 | 69.21 |
| R25 | 13 | 99.4 | 95 | 72.79 |
| R26 | 13 | 99.42 | 95.47 | 74.72 |
| R27 | 12 | 99.37 | 93.82 | 66.75 |
| R28 | 9 | 99.19 | 88.53 | 47.26 |
| R29 | 9 | 99.18 | 87.98 | 45.5 |
| R30 | 10 | 99.2 | 89.15 | 50.37 |
| R31 | 11 | 99.35 | 93.35 | 64.54 |
| R32 | 10 | 99.29 | 91.49 | 56.84 |
| R33 | 14 | 99.47 | 96.47 | 80.54 |
| R34 | 12 | 99.36 | 93.68 | 66.22 |
| R35 | 10 | 99.23 | 89.97 | 52.09 |
| R36 | 11 | 99.29 | 92.26 | 60.19 |
| R37 | 10 | 99.22 | 90.42 | 53.43 |
| R38 | 10 | 99.28 | 91.43 | 57.39 |
| R39 | 11 | 99.33 | 92.82 | 62.43 |
| R40 | 12 | 99.36 | 94.16 | 67.67 |
| R41 | 11 | 99.31 | 92.76 | 62.07 |
| R42 | 11 | 99.31 | 92.73 | 61.92 |
| R43 | 11 | 99.37 | 93.75 | 65.5 |
| R44 | 11 | 99.35 | 93.09 | 62.51 |
| R45 | 13 | 99.44 | 95.44 | 74.1 |
| R46 | 9 | 99.23 | 89.28 | 48.97 |
| R47 | 12 | 99.36 | 93.8 | 66.56 |
| R48 | 14 | 99.46 | 96.3 | 79.45 |
| R49 | 12 | 99.4 | 94.18 | 67.24 |
| R50 | 15 | 99.56 | 96.88 | 82.25 |
| R51 | 10 | 99.35 | 91.03 | 55.27 |
| R52 | 11 | 99.32 | 92.36 | 60.47 |
| R53 | 10 | 99.29 | 91.45 | 56.57 |
| R54 | 10 | 99.22 | 89.77 | 51.78 |
| R55 | 8 | 99 | 84.71 | 39.76 |
| R56 | 11 | 99.37 | 93.33 | 63.4 |
| R57 | 11 | 99.34 | 93.31 | 63.81 |
| R58 | 14 | 99.48 | 96.24 | 78.98 |
| R59 | 10 | 99.31 | 91.77 | 57.61 |
| R60 | 13 | 99.45 | 95.91 | 76.75 |
| R61 | 10 | 99.3 | 91.82 | 58.23 |
| R62 | 12 | 99.36 | 93.66 | 66.2 |
| R63 | 12 | 99.39 | 93.98 | 67.25 |
| R64 | 8 | 99.02 | 84.93 | 39.38 |
| R65 | 11 | 99.31 | 92.62 | 61.87 |
| R66 | 11 | 99.34 | 92.19 | 59.19 |
| R67 | 13 | 99.43 | 95.62 | 75.21 |
| R68 | 12 | 99.39 | 94.39 | 69.04 |
| R69 | 10 | 99.26 | 90.36 | 53.07 |
| R70 | 9 | 99.17 | 88.93 | 49.28 |
| R71 | 9 | 99.08 | 85.89 | 41.01 |
| R72 | 10 | 99.35 | 90.31 | 52.5 |
| R73 | 9 | 99.03 | 85.6 | 41.64 |
| R74 | 11 | 99.29 | 91.99 | 59.64 |
| R75 | 10 | 99.26 | 90.61 | 53.73 |
| R76 | 12 | 99.4 | 94.88 | 71.73 |
| R77 | 10 | 99.27 | 91.28 | 56.84 |
| R78 | 15 | 99.55 | 97.34 | 85.28 |
| R79 | 15 | 99.51 | 96.87 | 82.34 |
| R80 | 14 | 99.51 | 96.92 | 82.35 |
| R81 | 17 | 99.55 | 97.96 | 89.51 |
| R82 | 11 | 99.4 | 93.84 | 65.87 |
| R83 | 13 | 99.49 | 95.9 | 76.3 |
| R84 | 12 | 99.41 | 94.54 | 69.08 |
| R85 | 12 | 99.4 | 94.07 | 67.67 |
| R86 | 16 | 99.52 | 97.34 | 85.63 |
| R87 | 18 | 99.59 | 98.18 | 90.7 |
| R88 | 18 | 99.54 | 98.15 | 90.95 |
| R89 | 13 | 99.48 | 95.87 | 76.53 |
| R90 | 15 | 99.51 | 96.74 | 81.83 |
| R91 | 13 | 99.43 | 95.09 | 72.38 |
| R92 | 10 | 99.28 | 90.56 | 53.63 |
| R93 | 13 | 99.47 | 95.86 | 76.41 |
| R94 | 18 | 99.54 | 98.08 | 90.5 |
| R95 | 9 | 99.29 | 89.72 | 49.41 |
| R96 | 14 | 99.47 | 96.58 | 81.09 |
| R97 | 10 | 99.27 | 89.92 | 50.31 |
| R98 | 9 | 99.16 | 87.47 | 44.34 |
| R99 | 12 | 99.42 | 94.13 | 67.16 |
| R100 | 8 | 99 | 82.28 | 32.26 |
| R101 | 8 | 99.06 | 84.52 | 37.75 |
| R102 | 9 | 99.17 | 87.64 | 45.5 |
| R103 | 12 | 99.46 | 95.02 | 70.49 |
| R104 | 13 | 99.47 | 95.69 | 74.64 |
| R105 | 13 | 99.51 | 96.48 | 78.84 |
| R106 | 11 | 99.38 | 93.46 | 63.01 |
| R107 | 9 | 99.16 | 88.28 | 47.27 |
| R108 | 9 | 99.26 | 88.83 | 46.7 |
| R109 | 11 | 99.36 | 92.24 | 58.76 |
| R110 | 12 | 99.45 | 94.61 | 69.24 |
| R111 | 10 | 99.32 | 90.54 | 51.44 |
| R112 | 11 | 99.41 | 93.96 | 64.69 |
| R113 | 8 | 99.02 | 81.29 | 31.51 |
| R114 | 11 | 99.37 | 92.73 | 60.68 |
| R115 | 9 | 99.19 | 87.87 | 44.42 |
| R116 | 12 | 99.45 | 94.73 | 68.67 |
| R117 | 11 | 99.42 | 93.41 | 61.85 |
| R118 | 10 | 98.23 | 85.5 | 54.88 |
| R119 | 9 | 99.2 | 86.88 | 42.06 |
| R120 | 8 | 99.03 | 83.55 | 39.67 |
| R121 | 9 | 99.23 | 88.54 | 46.32 |
| R122 | 10 | 99.31 | 90.41 | 51.32 |
| R123 | 10 | 99.38 | 92.29 | 57.78 |
| R124 | 11 | 99.44 | 94.03 | 65.21 |
| R125 | 11 | 99.38 | 91.62 | 59.35 |
| R126 | 8 | 99.08 | 83.75 | 38.91 |
| R127 | 11 | 98.25 | 85.92 | 55.74 |
| R128 | 10 | 99.31 | 90.49 | 52.2 |
| R129 | 10 | 99.35 | 91.24 | 53.71 |
| R130 | 11 | 99.36 | 92.97 | 61.28 |
| R131 | 9 | 99.28 | 89.63 | 47.95 |
| R132 | 11 | 99.38 | 92.5 | 61.73 |
| R133 | 13 | 99.45 | 95.49 | 73.37 |
| R134 | 11 | 99.42 | 93.78 | 63.09 |
| R135 | 11 | 98.47 | 88.2 | 61.28 |
| R136 | 13 | 99.49 | 95.96 | 75.92 |
| R137 | 12 | 99.47 | 95.26 | 72.06 |
| R138 | 11 | 99.44 | 94.29 | 66.12 |
| R139 | 14 | 99.51 | 96.82 | 81.19 |
| R140 | 10 | 99.35 | 92.17 | 58.33 |
| R141 | 11 | 99.4 | 93.44 | 62.73 |
| R142 | 11 | 99.42 | 93.63 | 63.36 |
| R143 | 12 | 99.37 | 94.21 | 69.51 |
| R144 | 10 | 99.29 | 90.8 | 52.39 |
| R145 | 12 | 99.45 | 94.97 | 70.34 |
| R146 | 9 | 99.27 | 89.17 | 47.95 |
| R147 | 13 | 99.51 | 96 | 76 |
| R148 | 12 | 99.46 | 94.94 | 70.59 |
| R149 | 11 | 99.39 | 93.79 | 65.04 |
| R150 | 12 | 99.45 | 94.88 | 70.17 |
| R151 | 10 | 99.39 | 92.18 | 58.19 |
| R152 | 10 | 99.32 | 91.14 | 55.06 |
| R153 | 10 | 99.32 | 91.24 | 55.51 |
| R154 | 9 | 99.18 | 86.77 | 42 |
| R155 | 11 | 99.39 | 93.03 | 61.06 |
| R156 | 10 | 99.39 | 92.14 | 57.61 |
| R157 | 11 | 99.41 | 94.18 | 66.08 |
| R158 | 13 | 99.6 | 96.28 | 77.96 |
| R159 | 11 | 99.39 | 93.38 | 63.14 |
| R160 | 13 | 99.47 | 95.36 | 73.07 |
| R161 | 10 | 99.25 | 90.35 | 51.72 |
| R162 | 10 | 99.36 | 91.23 | 53.8 |
| R163 | 10 | 99.32 | 90.84 | 52.89 |
| R164 | 12 | 99.41 | 94.18 | 66.76 |
| R165 | 8 | 98.98 | 82.67 | 36.52 |
| R166 | 10 | 99.4 | 92.32 | 57.93 |

Ave_depth represents the average sequencing depth, Cov_ratio_1X% represents 1x coverage, Cov_ratio_5X% represents 5x coverage, and Cov_ratio_10X% represents 10x coverage.

Table S3 The Mapping rate for 166 Shaoxing ducks.

|  | Mapping rate | | |
| --- | --- | --- | --- |
| ID | Total_clean reads | Mapped(%) | Properly_mapped(%) |
| R01 | 90125256 | 96.61 | 90.85 |
| R02 | 95479782 | 96.81 | 91.4 |
| R03 | 79584534 | 96.77 | 91.91 |
| R04 | 92241584 | 96.71 | 91.22 |
| R05 | 79916988 | 96.56 | 91.08 |
| R06 | 86801070 | 96.86 | 91.97 |
| R07 | 92814852 | 96.68 | 90.85 |
| R08 | 89696974 | 96.64 | 91.12 |
| R09 | 89204212 | 96.77 | 91.59 |
| R10 | 99389330 | 96.6 | 91.07 |
| R11 | 101256396 | 96.63 | 90.94 |
| R12 | 107224692 | 96.55 | 90.84 |
| R13 | 105207268 | 96.69 | 91.09 |
| R14 | 106181270 | 96.7 | 91.04 |
| R15 | 101865016 | 96.76 | 91.06 |
| R16 | 105867664 | 96.69 | 90.98 |
| R17 | 89416504 | 96.47 | 90.76 |
| R18 | 107062296 | 96.88 | 91.41 |
| R19 | 123480444 | 96.62 | 90.91 |
| R20 | 100257540 | 96.77 | 91.09 |
| R21 | 109800952 | 96.76 | 91.23 |
| R22 | 111203866 | 96.59 | 91.17 |
| R23 | 105295116 | 96.89 | 91.51 |
| R24 | 104599570 | 96.57 | 90.88 |
| R25 | 109880600 | 96.58 | 91.26 |
| R26 | 111491294 | 96.75 | 91.1 |
| R27 | 100349110 | 96.74 | 91.26 |
| R28 | 81208596 | 96.52 | 90.81 |
| R29 | 78739522 | 96.6 | 91.08 |
| R30 | 81891676 | 96.95 | 91.58 |
| R31 | 96255164 | 96.94 | 91.66 |
| R32 | 89202006 | 96.7 | 91.32 |
| R33 | 121080758 | 96.81 | 91.55 |
| R34 | 100140902 | 96.64 | 91.06 |
| R35 | 84659676 | 96.64 | 91.3 |
| R36 | 92610108 | 96.7 | 91.17 |
| R37 | 84928644 | 96.72 | 91.64 |
| R38 | 89180220 | 96.83 | 91.69 |
| R39 | 95284184 | 96.72 | 91.34 |
| R40 | 100384412 | 96.79 | 91.43 |
| R41 | 94637676 | 96.63 | 91.1 |
| R42 | 93724712 | 96.87 | 91.63 |
| R43 | 98708588 | 96.65 | 90.94 |
| R44 | 96050124 | 96.62 | 90.89 |
| R45 | 108432436 | 96.93 | 91.78 |
| R46 | 81701680 | 96.77 | 91.25 |
| R47 | 99745966 | 96.65 | 91.36 |
| R48 | 121912104 | 96.45 | 90.83 |
| R49 | 99975860 | 96.7 | 91.67 |
| R50 | 125281116 | 96.38 | 90.98 |
| R51 | 87298900 | 96.73 | 91.64 |
| R52 | 92654260 | 96.73 | 91.44 |
| R53 | 88447800 | 96.79 | 91.56 |
| R54 | 84865916 | 96.67 | 91.25 |
| R55 | 74362056 | 96.6 | 90.96 |
| R56 | 95169024 | 96.79 | 91.38 |
| R57 | 97605774 | 96.58 | 90.88 |
| R58 | 119411040 | 96.61 | 90.99 |
| R59 | 87788078 | 96.89 | 92.26 |
| R60 | 114684420 | 96.68 | 90.95 |
| R61 | 89267622 | 96.77 | 91.88 |
| R62 | 99775002 | 96.68 | 91.4 |
| R63 | 100963868 | 96.53 | 91.11 |
| R64 | 73496160 | 96.62 | 91.32 |
| R65 | 93946694 | 96.62 | 91.59 |
| R66 | 91626212 | 96.63 | 91.09 |
| R67 | 113912080 | 96.39 | 90.67 |
| R68 | 102922920 | 96.7 | 91.2 |
| R69 | 86128848 | 96.66 | 90.96 |
| R70 | 82429992 | 96.65 | 91.21 |
| R71 | 74387186 | 96.82 | 91.54 |
| R72 | 86120342 | 96.51 | 90.97 |
| R73 | 76144698 | 96.64 | 90.84 |
| R74 | 92652262 | 96.46 | 91.34 |
| R75 | 86269916 | 96.83 | 91.76 |
| R76 | 104704938 | 96.89 | 92.15 |
| R77 | 89614340 | 96.64 | 91.34 |
| R78 | 137675628 | 96.26 | 89.27 |
| R79 | 130700946 | 96.1 | 88.75 |
| R80 | 125425216 | 96.44 | 90.4 |
| R81 | 148384412 | 96.4 | 90.49 |
| R82 | 100759074 | 96.36 | 90.22 |
| R83 | 117710456 | 96.24 | 89.84 |
| R84 | 104397774 | 96.37 | 90.22 |
| R85 | 105354098 | 96.12 | 89.48 |
| R86 | 137670840 | 96.37 | 89.98 |
| R87 | 173478154 | 96.07 | 86.9 |
| R88 | 156904788 | 96.28 | 90.04 |
| R89 | 119029962 | 96.16 | 89.52 |
| R90 | 126524664 | 96.44 | 90.44 |
| R91 | 110578278 | 96.22 | 89.63 |
| R92 | 87908564 | 96.32 | 89.67 |
| R93 | 115376538 | 96.33 | 89.87 |
| R94 | 158187348 | 96.18 | 89.74 |
| R95 | 85162666 | 96.23 | 89.65 |
| R96 | 131261354 | 95.87 | 89.04 |
| R97 | 85062542 | 96.4 | 89.92 |
| R98 | 79926970 | 96.32 | 89.54 |
| R99 | 102573016 | 96.59 | 90.28 |
| R100 | 69456014 | 96.23 | 89.57 |
| R101 | 75674136 | 96.18 | 89.27 |
| R102 | 80739048 | 96.41 | 90.05 |
| R103 | 104833244 | 96.23 | 90.01 |
| R104 | 112608162 | 96.1 | 89.28 |
| R105 | 116703068 | 96.45 | 90.43 |
| R106 | 97348974 | 96.33 | 90.04 |
| R107 | 82321970 | 96.18 | 89.06 |
| R108 | 82178570 | 96.25 | 89.63 |
| R109 | 98596116 | 96.01 | 88.11 |
| R110 | 105354402 | 96.25 | 89.9 |
| R111 | 86168068 | 96.1 | 89.57 |
| R112 | 97924464 | 96.48 | 90.51 |
| R113 | 86117918 | 95.26 | 82.39 |
| R114 | 96979570 | 96.32 | 89.32 |
| R115 | 80913908 | 96.05 | 89.13 |
| R116 | 102640962 | 96.3 | 89.91 |
| R117 | 93714134 | 96.5 | 90.42 |
| R118 | 92779910 | 96.79 | 92.72 |
| R119 | 78192822 | 96.09 | 89.75 |
| R120 | 79582916 | 95.62 | 88.45 |
| R121 | 80391508 | 96.3 | 90.27 |
| R122 | 85752672 | 96.3 | 89.93 |
| R123 | 92112994 | 96.32 | 89.53 |
| R124 | 98160284 | 96.32 | 89.85 |
| R125 | 101532364 | 95.71 | 88.06 |
| R126 | 95943354 | 95.37 | 82.41 |
| R127 | 95880246 | 96.78 | 92.31 |
| R128 | 86941370 | 96.35 | 89.82 |
| R129 | 86985246 | 96.42 | 89.55 |
| R130 | 96157498 | 96.08 | 89.09 |
| R131 | 83469352 | 96.38 | 89.13 |
| R132 | 101120356 | 95.81 | 88.96 |
| R133 | 109875520 | 96.26 | 89.44 |
| R134 | 97036266 | 96.31 | 89.78 |
| R135 | 103621938 | 96.63 | 92.45 |
| R136 | 114036160 | 96.29 | 89.87 |
| R137 | 107734142 | 96.12 | 89.6 |
| R138 | 99813050 | 96.37 | 89.53 |
| R139 | 122524568 | 96.3 | 89.5 |
| R140 | 92741504 | 96.18 | 89.76 |
| R141 | 96567778 | 96.34 | 89.64 |
| R142 | 96019620 | 96.45 | 89.94 |
| R143 | 104143504 | 96.25 | 89.16 |
| R144 | 86199912 | 96.34 | 89.66 |
| R145 | 106177146 | 96.28 | 89.46 |
| R146 | 84606760 | 96.01 | 88.92 |
| R147 | 114418814 | 96.04 | 89.53 |
| R148 | 109850752 | 96.07 | 89.3 |
| R149 | 99258854 | 96.31 | 90.02 |
| R150 | 107188124 | 96.08 | 89.23 |
| R151 | 95595484 | 95.92 | 88.85 |
| R152 | 90712596 | 96.06 | 89.58 |
| R153 | 90633498 | 96.31 | 89.83 |
| R154 | 77798728 | 96.12 | 89.65 |
| R155 | 98892974 | 96.16 | 88.44 |
| R156 | 107324126 | 95.75 | 85.03 |
| R157 | 99821312 | 96.39 | 89.9 |
| R158 | 117001880 | 96.4 | 90.15 |
| R159 | 96957398 | 96.4 | 90.22 |
| R160 | 109567184 | 96.42 | 90.29 |
| R161 | 89027132 | 96.39 | 88.95 |
| R162 | 88501686 | 96.08 | 89.54 |
| R163 | 88225802 | 96.04 | 88.84 |
| R164 | 102926124 | 96.19 | 89.57 |
| R165 | 79377898 | 95.91 | 87.07 |
| R166 | 92171508 | 96.14 | 89.39 |

Mapped (%) indicates the mapping rate, Properly Mapped (%) indicates the proper mapping rate.

**Table S4** Summary of the promising regions associated with LMW.

| Traits | chr | position | P_wald | R2 |
| --- | --- | --- | --- | --- |
| LMW | 1 | 137437762 | 4.34E-12 | 1 |
|  | 1 | 137432685 | 3.68E-07 | 0.796086 |
|  | 1 | 137439122 | 7.00E-06 | 0.784373 |
|  | 1 | 137437832 | 7.00E-06 | 0.784373 |
|  | 1 | 137436816 | 7.00E-06 | 0.784373 |
|  | 1 | 137432971 | 7.00E-06 | 0.784373 |
|  | 1 | 137432936 | 7.00E-06 | 0.784373 |
|  | 1 | 137432918 | 7.00E-06 | 0.784373 |
|  | 1 | 137437990 | 6.53E-06 | 0.784314 |
|  | 1 | 137433313 | 7.47E-06 | 0.784314 |
|  | 1 | 137433057 | 6.72E-06 | 0.784314 |
|  | 1 | 137433933 | 7.26E-06 | 0.784254 |
|  | 1 | 137433916 | 7.26E-06 | 0.784254 |
|  | 1 | 137432539 | 2.90E-06 | 0.770167 |
|  | 1 | 137432441 | 6.18E-07 | 0.723724 |
|  | 1 | 137432480 | 9.05E-07 | 0.723654 |
|  | 1 | 137494191 | 6.34E-06 | 0.711241 |
|  | 1 | 137487949 | 6.34E-06 | 0.711241 |
|  | 1 | 137487306 | 6.34E-06 | 0.711241 |
|  | 1 | 137432985 | 2.99E-06 | 0.711241 |
|  | 1 | 137487829 | 6.57E-06 | 0.711166 |
|  | 1 | 137487652 | 5.78E-06 | 0.711166 |
|  | 1 | 137438851 | 8.74E-05 | 0.707988 |
|  | 1 | 137438845 | 8.74E-05 | 0.707988 |
|  | 1 | 137437355 | 1.02E-04 | 0.707988 |
|  | 1 | 137433337 | 2.74E-05 | 0.701357 |
|  | 1 | 139279244 | 2.25E-08 | 0.690625 |
|  | 1 | 137437418 | 4.34E-04 | 0.672263 |
|  | 1 | 137438944 | 9.99E-05 | 0.649899 |
|  | 1 | 137438603 | 8.79E-04 | 0.649804 |
|  | 1 | 137488106 | 5.11E-07 | 0.639339 |
|  | 1 | 137479414 | 2.29E-06 | 0.639339 |
|  | 1 | 137488255 | 1.21E-04 | 0.635139 |
|  | 1 | 137487329 | 2.52E-04 | 0.635139 |
|  | 1 | 137439595 | 8.61E-05 | 0.635139 |
|  | 1 | 137439937 | 7.03E-05 | 0.633584 |
|  | 1 | 139276950 | 3.80E-10 | 0.630983 |
|  | 1 | 137523359 | 8.88E-05 | 0.627175 |
|  | 1 | 137513244 | 4.06E-06 | 0.627175 |
|  | 1 | 137412150 | 1.86E-03 | 0.589533 |
|  | 1 | 137463114 | 2.52E-04 | 0.578353 |
|  | 1 | 137433542 | 9.25E-05 | 0.578353 |
|  | 1 | 139331365 | 2.38E-07 | 0.568797 |
|  | 1 | 139301090 | 2.38E-07 | 0.568797 |
|  | 1 | 139292674 | 2.38E-07 | 0.568797 |
|  | 1 | 139279038 | 2.38E-07 | 0.568797 |
|  | 1 | 139262903 | 2.38E-07 | 0.568797 |
|  | 1 | 139251394 | 2.38E-07 | 0.568797 |
|  | 1 | 139251381 | 2.38E-07 | 0.568797 |
|  | 1 | 139249130 | 2.38E-07 | 0.568797 |
|  | 1 | 139247197 | 2.38E-07 | 0.568797 |
|  | 1 | 139293680 | 3.31E-08 | 0.568706 |
|  | 1 | 139267190 | 2.20E-07 | 0.568706 |
|  | 1 | 139261803 | 2.25E-07 | 0.568706 |
|  | 1 | 139256743 | 2.06E-07 | 0.568706 |
|  | 1 | 139247103 | 2.46E-07 | 0.568706 |
|  | 1 | 139255327 | 2.49E-07 | 0.568613 |
|  | 1 | 139255672 | 2.34E-07 | 0.56852 |
|  | 1 | 139255424 | 2.66E-06 | 0.559553 |
|  | 1 | 137432232 | 9.10E-03 | 0.554048 |
|  | 1 | 137432222 | 9.10E-03 | 0.554048 |
|  | 1 | 137432215 | 9.10E-03 | 0.554048 |
|  | 1 | 137422454 | 9.10E-03 | 0.554048 |
|  | 1 | 137422421 | 9.10E-03 | 0.554048 |
|  | 1 | 137440685 | 8.90E-03 | 0.553938 |
|  | 1 | 137428632 | 8.61E-03 | 0.553938 |
|  | 1 | 139238151 | 2.01E-07 | 0.533615 |
|  | 1 | 137432453 | 5.52E-04 | 0.533615 |
|  | 1 | 137490058 | 2.13E-03 | 0.530319 |
|  | 1 | 139239880 | 4.23E-07 | 0.522169 |
|  | 1 | 137432375 | 1.24E-02 | 0.50793 |
|  | 1 | 137432289 | 2.61E-02 | 0.50793 |
|  | 1 | 137429885 | 2.28E-02 | 0.50793 |
|  | 1 | 137428734 | 2.80E-02 | 0.507817 |
|  | 1 | 137427056 | 2.62E-02 | 0.507586 |
|  | 1 | 137427094 | 2.53E-02 | 0.507468 |
|  | 1 | 137427074 | 2.53E-02 | 0.507468 |
|  | 1 | 139269796 | 1.99E-07 | 0.499864 |
|  | 1 | 139256948 | 2.70E-08 | 0.499864 |
|  | 1 | 139255969 | 2.70E-08 | 0.499864 |
|  | 1 | 139249357 | 3.39E-09 | 0.499864 |
|  | 1 | 139276997 | 2.87E-08 | 0.499774 |
|  | 1 | 139250184 | 3.67E-08 | 0.499683 |
|  | 1 | 139256663 | 2.29E-05 | 0.492446 |
|  | 1 | 137423054 | 6.21E-02 | 0.492016 |
|  | 1 | 137422678 | 4.30E-02 | 0.492016 |
|  | 1 | 137427971 | 1.00E-02 | 0.487713 |
|  | 1 | 137422214 | 1.00E-01 | 0.487586 |
|  | 1 | 139332229 | 2.61E-07 | 0.481743 |
|  | 1 | 139332128 | 2.61E-07 | 0.481743 |
|  | 1 | 139294986 | 2.61E-07 | 0.481743 |
|  | 1 | 139292769 | 2.61E-07 | 0.481743 |
|  | 1 | 139245218 | 4.53E-07 | 0.481743 |
|  | 1 | 139240946 | 4.53E-07 | 0.481743 |
|  | 1 | 139232529 | 4.53E-07 | 0.481743 |
|  | 1 | 139217569 | 4.53E-07 | 0.481743 |
|  | 1 | 139216892 | 1.89E-07 | 0.481743 |
|  | 1 | 139332117 | 2.71E-07 | 0.481628 |
|  | 1 | 139327965 | 2.50E-07 | 0.481628 |
|  | 1 | 139326707 | 2.76E-07 | 0.481628 |
|  | 1 | 139301146 | 2.76E-07 | 0.481628 |
|  | 1 | 139294953 | 2.43E-07 | 0.481628 |
|  | 1 | 139238664 | 4.24E-07 | 0.481628 |
|  | 1 | 139236167 | 4.35E-07 | 0.481628 |
|  | 1 | 139235444 | 5.06E-07 | 0.481628 |
|  | 1 | 139234623 | 4.30E-07 | 0.481628 |
|  | 1 | 139238672 | 4.00E-07 | 0.481512 |
|  | 1 | 139236230 | 3.78E-07 | 0.481512 |
|  | 1 | 137432382 | 1.59E-02 | 0.475791 |
|  | 1 | 139331260 | 1.70E-05 | 0.475368 |
|  | 1 | 137407250 | 1.91E-02 | 0.461817 |
|  | 1 | 139238602 | 8.76E-08 | 0.444722 |
|  | 1 | 139234778 | 1.57E-07 | 0.444174 |
|  | 1 | 139297401 | 1.53E-05 | 0.438795 |
|  | 1 | 137492645 | 1.65E-02 | 0.438795 |
|  | 1 | 139268302 | 1.05E-08 | 0.433056 |
|  | 1 | 139267854 | 1.03E-08 | 0.432973 |
|  | 1 | 139330872 | 2.84E-04 | 0.42674 |
|  | 1 | 137431363 | 5.50E-02 | 0.422811 |
|  | 1 | 137520248 | 1.56E-03 | 0.422144 |
|  | 1 | 139227657 | 7.97E-07 | 0.416459 |
|  | 1 | 139226783 | 7.97E-07 | 0.416459 |
|  | 1 | 139225623 | 7.97E-07 | 0.416459 |
|  | 1 | 139225615 | 7.97E-07 | 0.416459 |
|  | 1 | 139223367 | 7.97E-07 | 0.416459 |
|  | 1 | 139220767 | 7.97E-07 | 0.416459 |
|  | 1 | 139227625 | 8.78E-07 | 0.416327 |
|  | 1 | 139220735 | 9.18E-07 | 0.416327 |
|  | 1 | 139294830 | 2.62E-07 | 0.410342 |
|  | 1 | 139293267 | 2.69E-07 | 0.410228 |
|  | 1 | 139236406 | 4.79E-07 | 0.410228 |
|  | 1 | 139297030 | 4.23E-08 | 0.409996 |
|  | 1 | 139120233 | 8.49E-06 | 0.402226 |
|  | 3 | 61180214 | 6.38E-04 | 1 |
|  | 3 | 61179349 | 2.88E-10 | 1 |
|  | 3 | 61134622 | 3.42E-10 | 1 |
|  | 3 | 61180892 | 2.18E-07 | 0.921986 |
|  | 3 | 61180124 | 2.18E-07 | 0.921986 |
|  | 3 | 61179381 | 2.18E-07 | 0.921986 |
|  | 3 | 61152126 | 2.18E-07 | 0.921986 |
|  | 3 | 61150412 | 2.18E-07 | 0.921986 |
|  | 3 | 61137711 | 2.18E-07 | 0.921986 |
|  | 3 | 61137585 | 2.18E-07 | 0.921986 |
|  | 3 | 61181960 | 2.06E-07 | 0.921961 |
|  | 3 | 61181021 | 2.90E-07 | 0.921935 |
|  | 3 | 61169532 | 2.63E-07 | 0.921935 |
|  | 3 | 61134603 | 1.98E-07 | 0.921935 |
|  | 3 | 61205342 | 2.91E-07 | 0.835998 |
|  | 3 | 61180035 | 2.39E-08 | 0.835998 |
|  | 3 | 61134709 | 2.91E-07 | 0.835998 |
|  | 3 | 61180026 | 2.84E-08 | 0.835948 |
|  | 3 | 61179992 | 2.84E-08 | 0.835948 |
|  | 3 | 61178769 | 1.37E-05 | 0.832171 |
|  | 3 | 61169546 | 1.59E-05 | 0.832066 |
|  | 3 | 61151082 | 3.50E-07 | 0.824468 |
|  | 3 | 61148413 | 3.12E-07 | 0.824411 |
|  | 3 | 61076572 | 2.73E-06 | 0.763977 |
|  | 3 | 61055615 | 6.15E-06 | 0.763977 |
|  | 3 | 61043480 | 2.73E-06 | 0.763977 |
|  | 3 | 61076552 | 2.75E-06 | 0.763902 |
|  | 3 | 61182757 | 1.70E-03 | 0.751462 |
|  | 3 | 61192806 | 2.77E-07 | 0.751396 |
|  | 3 | 61212549 | 2.35E-05 | 0.746521 |
|  | 3 | 61055224 | 1.19E-05 | 0.746307 |
|  | 3 | 61055536 | 1.08E-05 | 0.679462 |
|  | 3 | 61152025 | 2.39E-03 | 0.482236 |
|  | 3 | 60953189 | 4.94E-04 | 0.482124 |
|  | 3 | 60953180 | 4.94E-04 | 0.482124 |
|  | 3 | 61213423 | 1.70E-07 | 0.477798 |
|  | 3 | 61182939 | 5.04E-06 | 0.426181 |
|  | 3 | 61182917 | 4.91E-06 | 0.426181 |
|  | 3 | 61227243 | 1.81E-06 | 0.415156 |
|  | 3 | 61226853 | 6.48E-05 | 0.409024 |
|  | 3 | 61007084 | 1.92E-04 | 0.407064 |
|  | 3 | 60944962 | 6.15E-04 | 0.407064 |
|  | 3 | 60933263 | 1.35E-03 | 0.407064 |
|  | 3 | 60932650 | 1.35E-03 | 0.407064 |
|  | 3 | 60924904 | 1.35E-03 | 0.407064 |
|  | 3 | 60912335 | 1.35E-03 | 0.407064 |
|  | 3 | 61007105 | 1.88E-04 | 0.406928 |
|  | 3 | 60918779 | 1.29E-03 | 0.406928 |
|  | 3 | 60933922 | 1.29E-03 | 0.406791 |
|  | 3 | 60933019 | 1.56E-03 | 0.406791 |

P_wald represents the p-value of the locus obtained from GWAS, and R2 indicates the degree of linkage disequilibrium between the locus and the most significant locus 1_137437762.
